# Discovery of a novel ROS-based signature for predicting prognosis and immunosuppressive tumor microenvironment in lung adenocarcinoma

**DOI:** 10.7150/jca.93975

**Published:** 2024-03-17

**Authors:** Yufeng Guo, Geyun Chang, Rui Wan, Xue Zhang, Zixiao Ma, Hua Bai, Jie Wang

**Affiliations:** 1 National Cancer Center/National Clinical Research Center for Cancer/Cancer Hospital, Chinese Academy of Medical Sciences and Peking Union Medical College, Beijing, China, 100021.; 2 CAMS Key Laboratory of Translational Research on Lung Cancer, State Key Laboratory of Molecular Oncology, Department of Medical Oncology, National Cancer Center/National Clinical Research Center for Cancer/Cancer Hospital, Chinese Academy of Medical Sciences and Peking Union Medical College, Beijing, China, 100021.; 3 Department of Thoracic Surgery, Peking University People's Hospital, Beijing, China, 100044.

**Keywords:** dendritic cells, immunotherapy, LUAD, ROS, tumor microenvironment.

## Abstract

The role of reactive oxygen species (ROS) is critical in the emergence and progression of lung adenocarcinoma (LUAD), affecting cell survival, proliferation, angiogenesis, and metastasis. Further investigations are needed to elucidate these effects' precise pathways and devise therapeutic approaches targeting ROS. Moreover, the expression pattern and clinical significance of the ROS-related genes in LUAD remain elusive. Through comprehensive analysis incorporating 1494 LUAD cases from The Cancer Genome Atlas, six Gene Expression Omnibus series, and a Chinese LUAD cohort, we identified a ROS-related signature with substantial predictive value in various LUAD patient cohorts. The ROS-related signature has demonstrated a significant negative relationship with antitumor immunity and dendritic cell maturation and activation. Moreover, The ROS-related signature showed predictive value on immunotherapy outcomes across multiple types of solid tumors, including LUAD. These findings reinforce the ROS-related signature as a predictive tool for LUAD and provide new insights into its link with antitumor immunity and immunotherapy efficacy. These results have implications for refining clinical assessments and tailoring immunotherapeutic strategies for patients with LUAD.

## Background

Lung adenocarcinoma (LUAD), which represents the predominant subtype of non-small cell lung cancer (NSCLC), is among the most frequently diagnosed cancers worldwide. It ranks as the leading cause of cancer-related deaths, accounting for approximately 2 million new cases and 1.76 million fatalities each year 1. Despite significant progress in treatment modalities such as targeted and immunotherapy, the 5-year survival rate for LUAD from 2010 to 2014 remained at 10% to 20% across most nations 2. Immunotherapies, which target the immune system to recognize and attack cancer cells, have transformed treatment strategies for diverse types of solid and haematologic malignancies 3. Immune checkpoint inhibitors (ICIs), including monoclonal antibodies against programmed cell death-1 (PD-1) and its ligand PD-L1, have provided up to 40% response rate in LUAD patients of advanced stages 4. Favorable long-term outcomes with ICIs are observed in LUAD with high PD-L1 expression 5, high T-effector- and interferon-gamma-associated gene expression 6, mismatch-repair deficiency 7, microsatellite instability 8, and high TMB 9. However, few patients experience long-term disease remission with immune checkpoint inhibitors (ICIs) 10. Thus, identifying novel biomarkers and developing a comprehensive understanding of underlying mechanisms of immunotherapy response in LUAD is imperative.

Reactive oxygen species (ROS), which include reactive molecules like superoxide anions (O2-) and hydroxide ions (OH-) 11, play pivotal roles in cellular signaling, impacting cell growth, differentiation, and proliferation 12. When there is a redox imbalance—where the generation of ROS outpaces a cell's ability to neutralize them—oxidative stress ensues 13. This surplus of ROS is implicated in several aspects of lung adenocarcinoma (LUAD) tumorigenesis. ROS can damage DNA, proteins, and lipids, which may result in genetic mutations, disrupted signaling pathways, and compromised cellular membranes, all of which can spur the onset and advancement of LUAD 14. Additionally, ROS can stimulate various signaling pathways that are key to cancer cell survival, growth, the formation of new blood vessels, and the spread of cancer cells 15. Emerging studies indicate that ROS might enhance the malignancy of LUAD and, in concert with the tumor microenvironment (TME), contribute to increased tumor aggression and resistance to treatment 16. Nonetheless, the literature has yet to fully elucidate the role of ROS in the anti-tumor immune response and the effectiveness of immunotherapy for LUAD.

Our research devised a predictive signature rooted in ROS-associated gene expression and LUAD patient data. This signature exhibited a strong correlation with patient outcomes within The Cancer Genome Atlas (TCGA)-LUAD cohort and six other independent cohorts. We also created a detailed nomogram for estimating LUAD overall survival (OS) probabilities. This ROS-based signature was linked to LUAD's tumor immune microenvironment (TIME). Further analysis into biological pathways and immune cell infiltration suggested that the signature's association with poor prognosis could be due to its dampening effect on dendritic cell (DC) maturation and activation. Comparisons with nine other recognized predictive gene signatures across various immunotherapy cohorts of multiple cancer types highlighted the distinctive predictive capabilities of the ROS-related signature. Pharmacological inhibition FOXM1—a gene within the ROS-related signature—proved effective in curbing tumor growth and boosting immunotherapy response in the LLC mouse model.

## Materials and Methods

### Publicly available mRNA data and the ROS-related geneset

**Figure 1:**
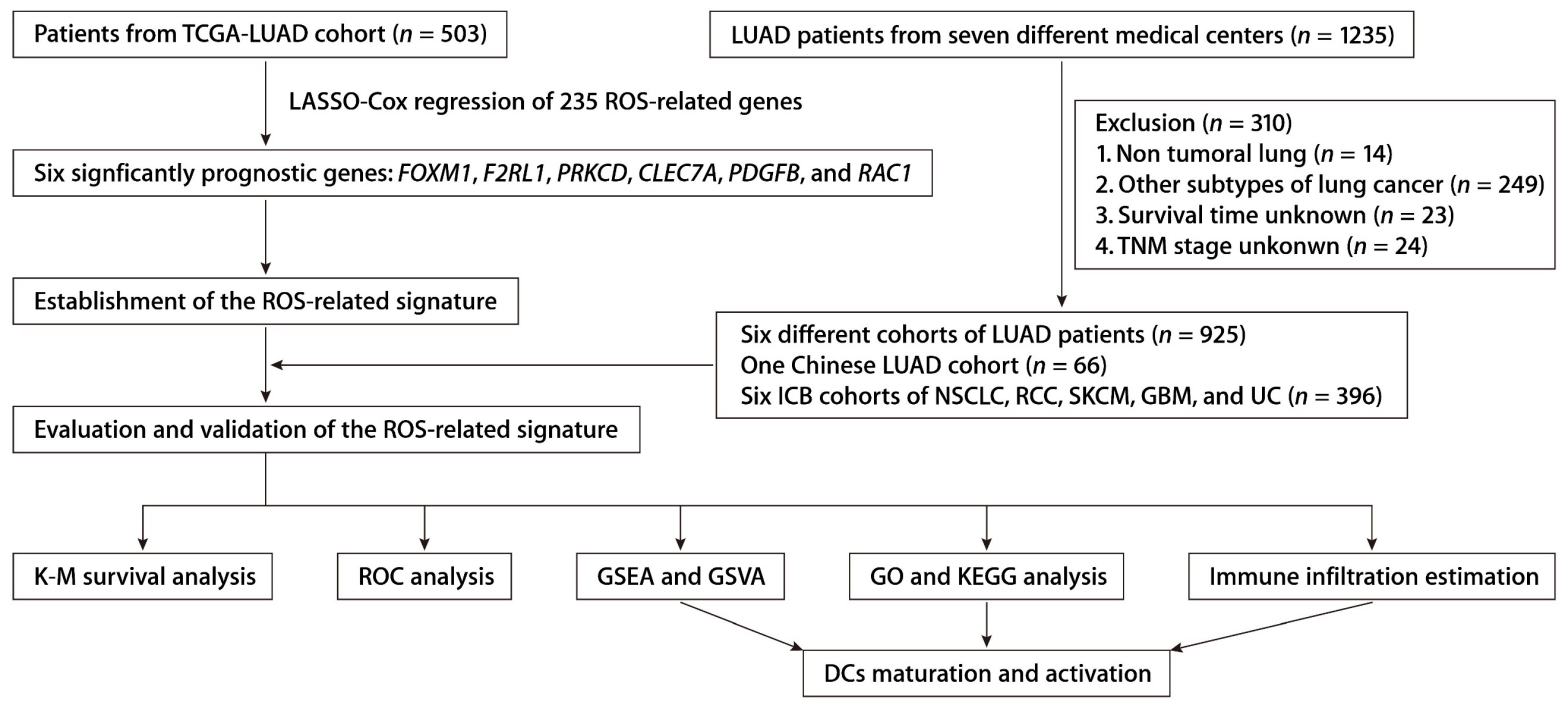
The workflow of establishing and validating the ROS-related gene signature in LUAD patients.

We integrated data from two publicly accessible sources. The workflow is depicted in Figure 1. We obtained TCGA-LUAD patient sample data from the UCSC Xena platform. Additional LUAD datasets were retrieved from Gene Expression Omnibus (GEO) series GSE50081, GSE13213, GSE30219, GSE72094, GSE29016, and GSE26939. The ROS-associated gene set (Table S1) was procured from the Molecular Signatures Database (MsigDB). We included the NSCLC anti-PD-1 cohort (GSE126044), the skin cutaneous melanoma (SKCM) anti-PD-1 cohort, the renal cell carcinoma (RCC) anti-PD-1 cohort, the glioblastoma (GBM) anti-PD-1 cohort, and the urothelial carcinoma (UC) anti-PD-L1 cohort to assess the predictive efficacy regarding immunotherapy response. Data from patients with incomplete information were excluded.

### Immune infiltration estimation

We conducted a single-sample gene set enrichment analysis (ssGSEA) on 28 immune-related gene sets, encompassing a range of genes pertinent to diverse immune cell types, their functions, pathways, and regulatory checkpoints, as referenced in 17. In particular, for assessing immune infiltration within LUAD, the ssGSEA method was applied using the 'GSVA' software from the R programming environment 17. This approach helped determine the presence of 28 distinct immune cell varieties and comprehensively evaluate the immune profile for each LUAD sample in the analysis. Additionally, the abundance of immune cells infiltrating the tumor and components of the stromal compartment were quantified using the 'MCPcounter' package for R 18.

### Analysis of differentially expressed genes (DEGs) and signature development

Prognostic genes were identified using the Cox proportional hazards model, and risk scores were computed using their expression levels. Based on the ROS-related signature expression, the LIMMA method was applied to discern DEGs between high- and low-risk LUAD patients.

### Risk stratification of patients in TCGA-LUAD cohort and GEO datasets

Risk stratification of LUAD patients was performed according to the ROS-related signature expression via the 'survivalROC' package 19 in R. Using Kaplan-Meier (K-M) survival curves and the log-rank test, we assessed the significance of the ROS-related signature in predicting prognosis in patients from TCGA-LUAD. The robustness of the signature was further corroborated through similar methodologies across a range of GEO datasets, such as GSE50081, GSE13213, GSE30219, GSE72094, GSE29016, and GSE26939. We also conducted univariate Cox regression analysis to contrast the impact of the signature with other clinical predictors. Furthermore, a predictive nomogram was developed, and its accuracy was validated through receiver operating characteristic (ROC) analysis and K-M survival estimates.

### Sample collection and panel RNA-seq of the Chinese LUAD validation cohort

We collected 66 frozen LUAD tissue samples from the National Cancer Center/Cancer Hospital, Chinese Academy of Medical Sciences, from May 2013 to September 2018. Total RNA extraction followed standard procedures using RNAiso Plus reagent (Takara). Subsequent panel RNA-seq was performed as per the manufacturer's guidelines. The study adhered to protocols sanctioned by the relevant ethics committee, and informed consent was secured from all patients. Event numbers for each category and variable in the Chinese cohort are detailed in Table S2, with metadata and RNA-seq in Tables S3 and S4, respectively.

### Functional enrichment analysis and gene set variation analysis (GSVA)

Functional enrichment for the Kyoto Encyclopedia of Genes and Genomes (KEGG) and Gene Ontology (GO) was conducted using the “clusterProfiler” package 20 in R, with significant pathways identified at *P* < 0.05. GSVA assessed signaling pathway changes across TCGA-LUAD samples. Gene Set Enrichment Analysis (GSEA) via “javaGSEA” software 21 between different risk groups was also conducted.

### Cell culture and gene silencing by lentiviral transduction

The syngeneic mouse lung cancer cell line, LLC, was cultivated under standard conditions and tested to ensure the absence of mycoplasma contamination. We performed gene silencing of *FOXM1* in these cells using a lentiviral system designed to express short-hairpin RNAs (shRNAs) (Table S5). Post-transduction selection with puromycin ensured the proliferation of only successfully transduced cells.

### Animal studies

We obtained C57BL/6J (C57) mice from Charles River Laboratories. In these 5-week-old C57 mice, we introduced xenografts by subcutaneously inoculating half a million cells. We recorded tumor expansion tri-weekly, utilizing a caliper to measure the perpendicular diameters. We computed tumor size in cubic millimeters using the formula (Length × Width^2^)/2, with Length and Width as the primary dimensions. The protocol necessitated humane euthanasia of the mice upon the principal tumor dimension attaining 15 mm. When tumor volume reached approximately 500 mm^3^, we removed and prepared them for subsequent flow cytometry. All procedural work with the mice conformed to the ethical guidelines set forth by the National Cancer Center/Cancer Hospital's ethics committee at the Chinese Academy of Medical Sciences.

### Immunohistochemistry (IHC) staining

LUAD tissue sections were prepared and incubated with rabbit monoclonal anti-FOXM1 (Abcam, ab207298) and mouse monoclonal anti-PD-L1 (Proteintech, 66248-1-Ig) diluted at a ratio of 1:500 and incubated at 4 degrees Celsius through the night. We conducted immunostaining using the Envision System and diaminobenzidine as the chromogen (sourced from Dako). We employed Image-Pro Plus version 6.0 software to evaluate the FOXM1 and PD-L1 expression.

### Statistical analysis

R software and GraphPad Prism were applied for statistical analyses. The Wilcoxon test compared immune cell infiltration, and survival data was analyzed using the log-rank test. *P* < 0.05 was considered statistically significant.

## Results

### Construction of a ROS-related predictive signature

**Figure 2:**
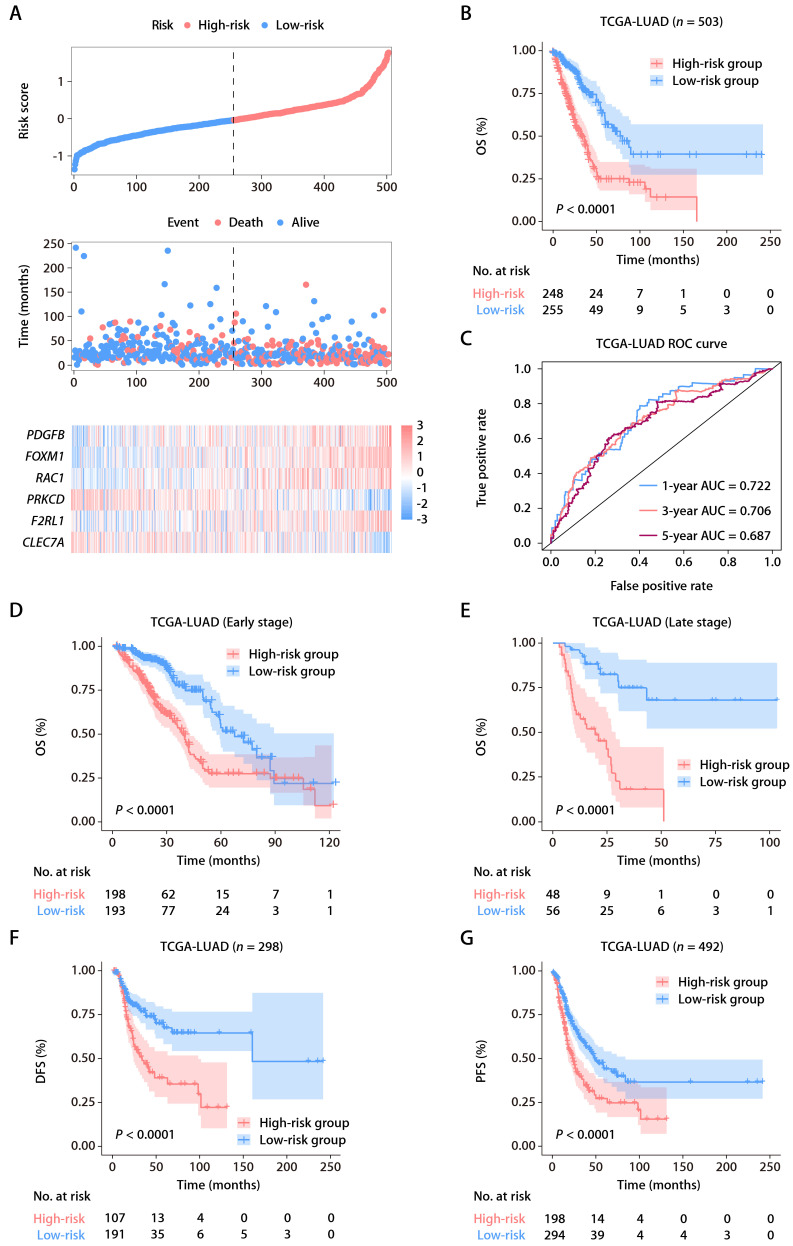
** Identification and establishment of the ROS-related signature. (A)** The distributions of the risk score, survival status, and gene expression panel. **(B)** K-M survival analysis of OS between the high- and low-risk groups stratified by the ROS-related signature in TCGA-LUAD cohort. **(C)** AUC values of ROC for predicting 1-, 3- and 5-year OS in patients in TCGA-LUAD cohort. **(D)** K-M survival analysis of OS between the high- and low-risk groups stratified by the ROS-related signature in TCGA-LUAD cohort with early-stage disease (stage I and II, *n* = 391). **(E)** K-M survival analysis of OS between the high- and low-risk groups stratified by the ROS-related signature in TCGA-LUAD cohort with advanced-stage disease (stage III and IV, *n* = 104). **(F)** K-M survival analysis of DFS between the high- and low-risk groups stratified by the ROS-related signature in TCGA-LUAD cohort. **(G)** K-M survival analysis of PFS between the high- and low-risk groups stratified by the ROS-related signature in TCGA-LUAD cohort.

From the MSigDB, we gathered 235 genes associated with ROS (Table S1). Using the “glmnet” package's least absolute shrinkage and selection operator (LASSO) technique, we pinpointed 13 ROS-related genes with differential expression and prognostic significance. A subsequent Cox proportional hazards regression analysis distilled this to six key predictive genes: *FOXM1*, *F2RL1*, *PRKCD*, *CLEC7A*, *PDGFB*, and *RAC1*. We derived a risk score formula: risk score = (0.15456 × *FOXM1*) + (0.17907 × *F2RL1*) + (-0.38644 × *PRKCD*) + (-0.16558 × *CLEC7A*) + (0.18365 × *PDGFB*) + (0.27251 × *RAC1*). Patient demographics and clinical features from TCGA-LUAD cohort are detailed in Table S2. The expression patterns of these six ROS-related genes were closely tied to risk scores, and an optimal cutoff was employed to categorize patients into high- or low-risk (Fig. 2A). K-M survival analysis demonstrated significantly poorer outcomes for the high-risk group (Fig. 2B). The predictive accuracy was gauged using time-dependent ROC curves, with the ROS-related signature showing AUCs of 0.722, 0.706, and 0.687 at 1, 3, and 5 years, respectively, within TCGA-LUAD cohort (Fig. 2C). As early-stage (clinical stages I and II) and advanced-stage (clinical stages III and IV) LUAD have different therapeutic approaches and prognoses 22, we also assessed the ROS-related signature in TCGA-LUAD patients across these stages. A higher risk score correlated with poorer OS in both early (Fig. 2D) and advanced stages (Fig. 2E). Additionally, the signature was a significant indicator for disease-free survival (DFS; Fig. 2F) and progression-free survival (PFS; Fig. 2G).

### Validation of the ROS-related signature in various LUAD cohorts

**Figure 3:**
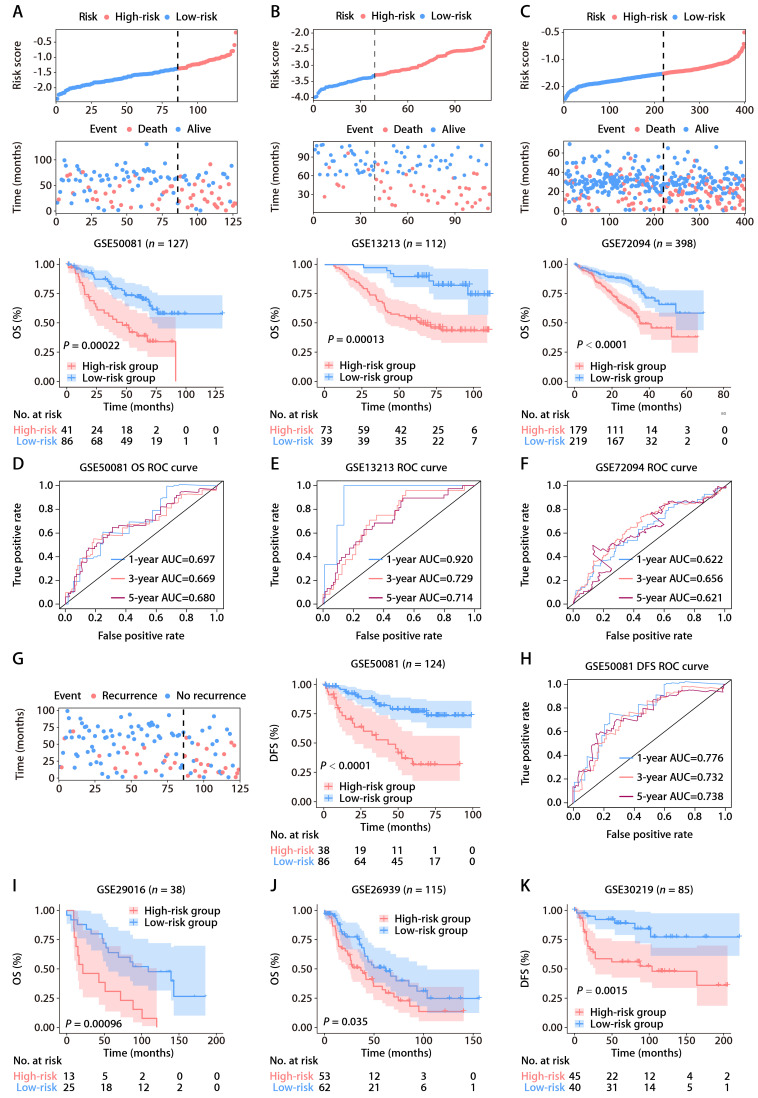
** Validation of the ROS-related signature in six independent cohorts. (A-C)** Distributions of the risk score and survival status (upper and middle panels) and K-M curves of OS between the high- and low-risk groups of LUAD patients (lower panel) in the GSE50081, GSE13213, and GSE72094 datasets. **(D-F)** AUC values of ROC for predicting 1-, 3- and 5-year OS in patients in the GSE50081, GSE13213, and GSE72094 cohorts. **(G)** The distributions of survival status (left panel) and K-M curves of DFS (right panel) between the high- and low-risk groups of LUAD patients in the GSE50081 dataset. **(H)** AUC values of ROC for predicting 1-, 3- and 5-year DFS in patients in the GSE50081 dataset. **(I-K)** K-M survival analysis of OS (GSE29016 and GSE26939) and DFS (GSE30219) between the high- and low-risk groups stratified by the ROS-related signature in three additional GEO datasets.

To affirm the consistency of the ROS-related signature for LUAD patients, we computed each patient's risk score in six distinct GEO datasets, utilizing the established formula. The patient demographics from these datasets are detailed in Table S2. Patients were categorized into high and low-risk groups according to the optimal threshold. K-M analyses showed that high-risk patients had poorer OS than those at low risk across the GSE50081 (Fig. 3A), GSE13213 (Fig. 3B), and GSE72094 (Fig. 3C) cohorts. Time-dependent ROC analyses further established the prognostic strength of the ROS-related signature, with the GSE50081 dataset exhibiting AUCs of 0.697, 0.669, and 0.680 for 1, 3, and 5-year OS (Fig. 3D). Comparable outcomes were noted for the GSE13213 and GSE72094 datasets, which displayed AUCs of 0.920, 0.729, and 0.714, and 0.622, 0.656, and 0.621, respectively (Fig. 3E and 3F). Moreover, significant DFS differences were observed between high and low-risk groups in the GSE50081 cohort (Fig. 3G), with respective 1, 3, and 5-year AUCs of 0.776, 0.732, and 0.738 (Fig. 3H). The ROS-related signature also effectively predicted OS and DFS in the GSE29016 (Fig. 3I), GSE26939 (Fig. 3J), and GSE30219 (Fig. 3K) datasets. These findings corroborate the signature's precision and reliability in predicting survival for LUAD patients. We further assessed the signature's stability across different clinical subgroups. Patients were sorted by risk score and divided into high and low-risk categories, with K-M analyses evaluating OS differences. The results consistently showed shorter OS in the high-risk group across all subgroups (Fig. S2A-S2F). Additionally, when assessing the signature's performance in subsets of patients with various mutations, we found that the ROS-based risk factor effectively differentiated OS in groups defined by *EGFR* wild-type (WT), *EGFR* mutation (MUT), *KRAS*^WT^, *KRAS*^MUT^, and *EGFR*^WT^/*KRAS*^WT^ statuses (Fig. S3A-S3E).

Both univariate and multivariate Cox regression analyses were conducted within TCGA-LUAD cohort to determine if the prognostic relevance of the ROS-based signature was independent of other clinical and pathological factors. These analyses showed that the risk score independently correlated with OS in LUAD patients, as detailed in Tables S6 and S7. This finding was replicated across six additional LUAD cohorts (Tables S6 and S7), reinforcing that the ROS-related signature possesses a greater capacity to differentiate between LUAD patients with varying prognoses than other clinicopathological features.

**Figure 4:**
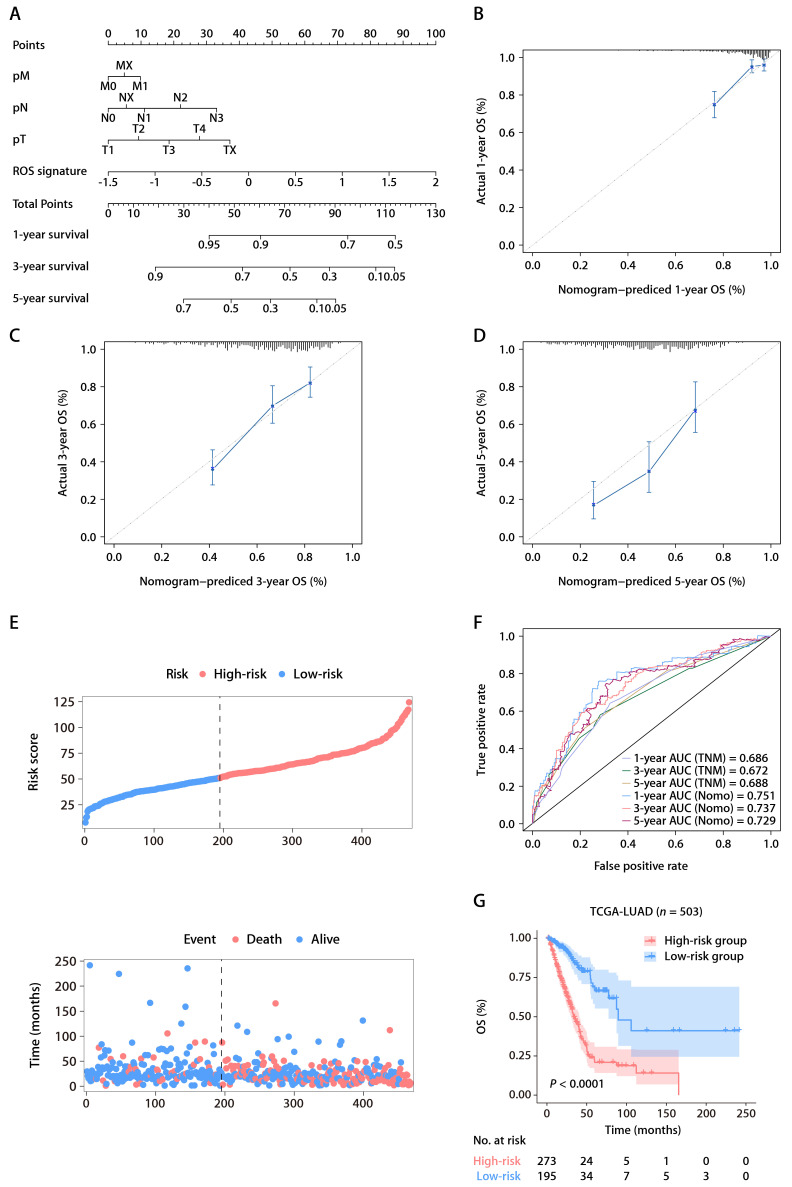
** The ROS-based nomogram for predicting OS in TCGA-LUAD cohort. (A)** Nomograms for predicting the 1-, 3-, and 5-year OS of patients in TCGA-LUAD cohort. **(B-D)** Calibration curves for predicting patient OS at 1 (B), 3 (C), and 5 years (D). **(E)** The distributions of the risk score calculated by the nomogram and survival status of TCGA-LUAD patients. **(F)** Time-dependent ROC analysis to assess the predictive ability of the nomogram and TNM staging system at 1, 3, and 5 years. **(G)** K-M survival analysis of OS between the high- and low-risk patients in TCGA-LUAD cohort.

Univariate Cox regression analysis within TCGA-LUAD cohort identified four predictors of patient outcomes, namely, the ROS-related signature, T stage, N stage, and M stage. These factors were used to construct a prognostic nomogram (Fig. 4A). The accuracy of the nomogram was assessed using calibration curves, which tested the agreement between the predicted and actual outcomes (Fig. 4B-D). The nomogram's predictions, along with patient survival and risk scores, are depicted in Figure 4E. Time-dependent ROC analysis was employed to evaluate the nomogram's predictive performance, yielding AUC values of 0.751, 0.737, and 0.729 for 1-, 3-, and 5-year OS, respectively (Fig. 4F). When compared with the TNM staging system, which is the current clinical standard, the gene signature demonstrated better predictive ability, as evidenced by the bootstrap method analysis (Fig. 4F). The nomogram stratified patients into low- and high-risk groups, with the former showing significantly better survival in the K-M analysis (Fig. 4G). Furthermore, a multivariate Cox regression analysis across various TCGA pan-cancer cohorts pinpointed the ROS-related signature as a significant prognostic indicator not just in LUAD but also in mesothelioma (MESO), melanoma (SKCM), head and neck cancer (HNSC), pancreatic cancer (PAAD), cervical cancer (CESC), adrenocortical cancer (ACC), lower-grade glioma (LGG), thyroid cancer (THCA), and kidney papillary cell carcinoma (KIRP) (Fig. S4). This suggests the ROS-related signature's broad applicability in predicting survival across multiple solid tumor types, underscoring its substantial translational potential.

### Functional annotation of the ROS-related signature

**Figure 5:**
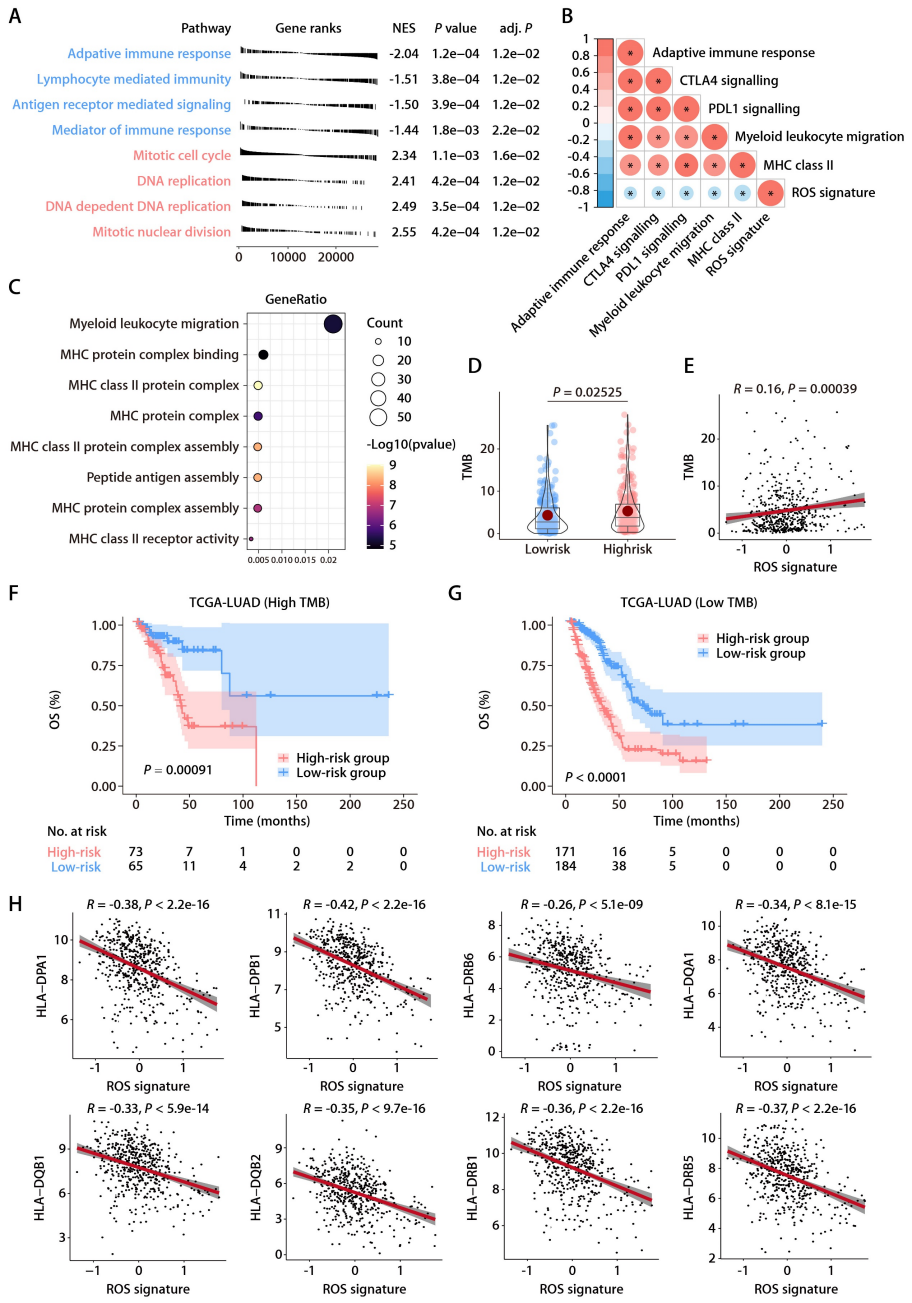
** Functional annotation of the ROS-related signature in LUAD. (A)** Significantly enriched gene sets based on the DEGs between the high- and low-risk groups in TCGA-LUAD cohort. **(B)** Correlation analysis between the risk score and immune metagenes. **(C)** GO annotation of the DEGs between the high- and low-risk groups. **(D)** The distributions of TMB between the high- and low-risk groups. **(E)** Correlation analysis between the risk score and TMB. **(F)** K-M survival analysis of OS between the high- and low-risk groups of patients with high TMB. **(G)** K-M survival analysis of OS between the high- and low-risk groups of patients with low TMB. **(H)** Correlation analysis between the risk score and HLA genes.

The robustness of the ROS-related signature in predicting the OS of patients with LUAD prompted further investigation into the biological pathways associated with the signature. Differential gene expression analysis between high- and low-risk groups identified significant DEGs using a threshold of an absolute log2 fold change greater than one and a *P* value less than 0.05. GSEA on these genes showed significant enrichment of downregulated genes in immune and antigen-related pathways and upregulation in DNA replication pathways, suggesting a link between the ROS-related signature and the TIME (Fig. 5A). Further immune pathway analysis indicated a negative correlation between the ROS-related signature and aspects of the immune response, such as myeloid leukocyte migration and the major histocompatibility complex (MHC)-II complex (Fig. 5B). GO annotations also showed a significant relationship between the signature and antigen presentation and MHC complexes (Fig. 5C). Additionally, the tumor mutational burden (TMB) that serves as an indicator of a tumor's immunogenicity, demonstrated a positive correlation with the ROS-related signature (Fig. 5D and 5E). The OS of LUAD patients with higher ROS-related signature expression was notably inferior to those with lower expression, irrespective of their TMB status (Fig. 5F and 5G). This highlights the signature's potential utility in predicting responses to LUAD treatments. Notably, the expression levels of specific HLA genes, including *HLA-DPA1*, *HLA-DPB1*, *HLA-DRB6*, *HLA-DQA1*, *HLA-DQB1*, *HLA-DQB2*, *HLA-DRB1*, and *HLA-DRB5*, had a negative association with the ROS-related signature (Fig. 5H). These findings underline the connection between the ROS-related signature and antigen presentation and antitumor immunity, which opens avenues for further exploration into the mechanisms by which the ROS-related signature could predict LUAD outcomes.

**Figure 6:**
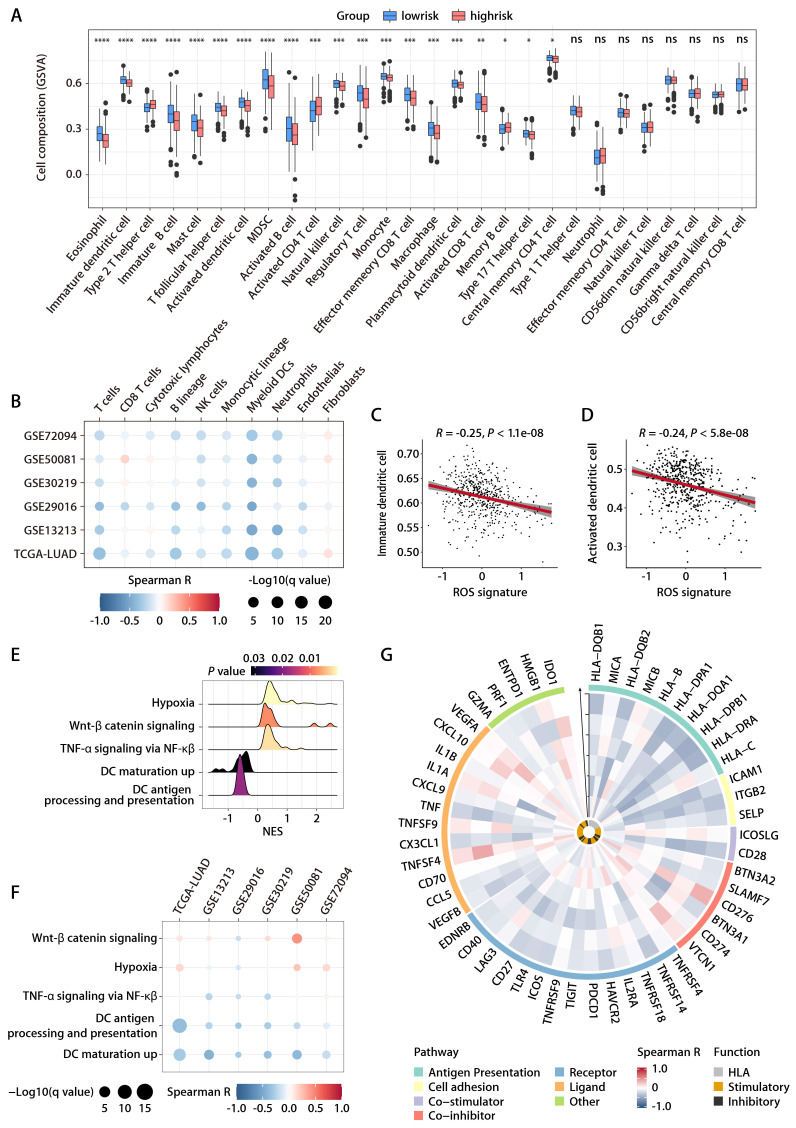
** Relationship between the ROS-related signature and antitumor immunity. (A)** Comparison of immune subpopulation infiltration inferred by ssGSEA between the high- and low-risk groups in TCGA-LUAD cohort. **(B)** Heatmap depicting the correlation between the risk score and the infiltration of immune cells inferred by MCP-counter in the training and validation cohorts. **(C and D)** Correlation analysis between the risk score and immature (C) or activated DCs (D). **(E)** GSEA of the DEGs between the high- and low-risk groups in TCGA-LUAD cohort. **(F)** Heatmap depicting the correlation between the risk score and significantly enriched gene sets in the training and validation cohorts. **(G)** Circos plot depicting the correlation between the risk score and the expression level of immune‐related genes in the training and validation cohorts. From inside to outside the Circos plot, the vertical axis with a black arrow indicates different LUAD cohorts, which are annotated on the y-axis in Figure 6B.

To elucidate the association between the ROS-related signature and the TIME in LUAD, the ssGSEA algorithm 17, leveraging 29 immune gene sets, was employed to quantify the infiltration levels of various immune cells in TCGA-LUAD cohort. High-risk patients exhibited a reduction in cytotoxic immune cells such as CD8^+^ T cells, NK cells, and macrophages (Fig. 6A). Additionally, the MCP-counter method 18 showed a negative link between the ROS-related signature and tumor-infiltrating leukocytes, with myeloid DCs most strongly inversely related to the signature (Fig. 6B). Notably, both immature DCs and activated DCs infiltrated more in the low-risk group, which corroborated the ssGSEA findings (Fig. 6A, 6C, and 6D), supporting the theory that the ROS-related signature inversely affects antitumor immunity. Furthermore, the analysis of hallmark pathways revealed that hypoxia, Wnt β-catenin signaling, and TNF-α signaling via NF-κβ—pathways known to undermine the immune response 23—were more active in high-risk group tumors (Fig. 6E). Conversely, pathways for DC maturation and antigen processing were more active in the low-risk group (Fig. 6E), aligning with the ssGSEA outcomes, with consistent findings across validation cohorts (Fig. 6F). An extensive analysis of the ROS-related signature against 75 immune-related genes 24 revealed a generally negative correlation with immune gene expression levels in both the training and validation cohorts (Fig. 6G). In particular, the signature was inversely related to HLA gene expression and the expression of the costimulatory molecule CD28, while it positively correlated with the expression of inhibitory molecules like CD276, VTCN1, VEGFA, and IDO1 (Fig. 6G). These findings suggest that low ROS-related signature expression indicates stronger antitumor immunity compared to high expression. It implies that the ROS-related signature could play a pivotal role in immune regulation within LUAD by influencing DC maturation and activation.

### Evaluation of the ROS-related signature in the Chinese LUAD cohort

**Figure 7:**
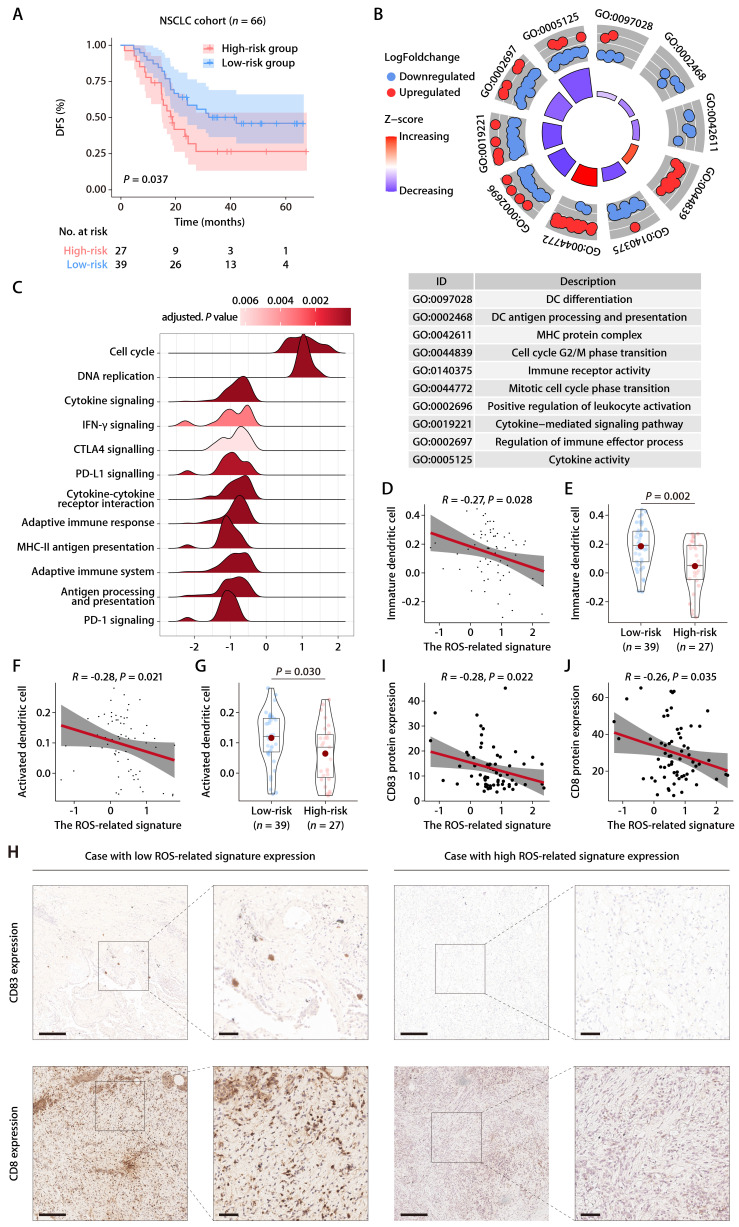
** Evaluation of the ROS-related signature in the Chinese LUAD cohort. (A)** K-M survival analysis of DFS between the high- and low-risk groups in the Chinese LUAD cohort. **(B)** GO annotation of the DEGs between the high- and low-risk groups in the Chinese LUAD cohort. **(C)** GSEA of the DEGs between the high- and low-risk groups in the Chinese LUAD cohort. **(D)** Correlation analysis between the risk score and immature DCs. **(E)** The distributions of immature DCs between the high- and low-risk groups. **(F)** Correlation analysis between the risk score and activated DCs. **(G)** The distributions of activated DCs between the high- and low-risk groups. **(H)** Representative microscopic images of CD83 and CD8 protein expression tested by IHC in tissue samples from patients of the Chinese LUAD cohort. The scale bars represent 200 µm (left panel) and 50 µm (right panel) in each case, respectively. **(I)** Correlation analysis between the ROS-related signature and CD83 protein expression quantified by IHC in tissue samples from patients of the Chinese LUAD cohort. **(J)** Correlation analysis between the ROS-related signature and CD8 protein expression quantified by IHC in tissue samples from patients of the Chinese LUAD cohort.

To rigorously evaluate the predictive capacity of the six-gene ROS-related signature for OS in LUAD patients in a clinical setting, the signature was further assessed in an independent cohort from China. This cohort included 66 LUAD patients, with each individual's risk score calculated using the expression levels of the six ROS-associated genes. Consistent with previous datasets, the Chinese LUAD cohort exhibited a marked difference in survival times, with the high-risk group showing significantly shorter PFS compared to the low-risk group (Fig. 7A). Cox proportional hazards regression analysis substantiated the risk score as a prominent prognostic indicator for patient outcomes in the Chinese LUAD cohort. The risk score consistently emerged as a robust prognostic factor in multivariate Cox regression analysis considering various datasets. The hazard ratios (HRs) and 95% confidence intervals (CIs) calculated underscored the stability of the risk score as an evaluation metric, detailed in Tables S6 and S7. Corroborating the findings from the TCGA-LUAD cohort, an inverse relationship was noted between the ROS-related signature and pathways associated with immunity, specifically those involving DC antigen processing and presentation, as well as the activity of MHC protein. This correlation extended to broader immune-related signaling, including immune receptor activity, leukocyte activation regulation, cytokine-mediated signaling, immune effector process regulation, and cytokine activity. These pathways were inversely associated with the ROS-related signature, while pathways related to cell proliferation exhibited a positive correlation (Fig. 7B). GO and KEGG functional enrichment analyses, along with immune metagene analyses, revealed that processes like MHC antigen presentation, adaptive immunity, cytokine-cytokine receptor interaction, and signaling pathways involving IFN-γ, CTLA-4, and PD-1/PD-L1 were significantly involved in the low-risk group (Fig. 7C). These insights indicate potential mechanisms by which DC activation may regulate antitumor immunity, particularly in tumors characterized by low ROS-related signature expression. Further investigation into the infiltration of DCs demonstrated a consistent negative association between the ROS-related signature and both immature and activated DC populations in the Chinese LUAD cohort (Fig. 7D-7G). This pattern aligns with the training and validation cohorts' findings. IHC analyses performed on the Chinese LUAD cohort tissues affirmed these associations at the protein level. Specifically, the ROS-related signature exhibited a significant negative correlation with CD83 expression, a protein highly and stably expressed by mature DCs upon activation 25-27 (Fig. 7H and 7I). This finding was in agreement with the transcriptomic data from the training and validation cohorts. Similarly, a negative correlation was found between the ROS-related signature and CD8 protein expression (Fig. 7H and 7J), which implies a possible involvement of the ROS-related signature in modulating T cell-mediated antitumor responses through its impact on DC maturation and activation. These multifaceted analyses provide a comprehensive view of the ROS-related signature's role in LUAD, highlighting its potential as a significant biomarker for patient stratification and prognosis in LUAD.

### Predictive value of the ROS-related signature on immunotherapy outcome

**Figure 8:**
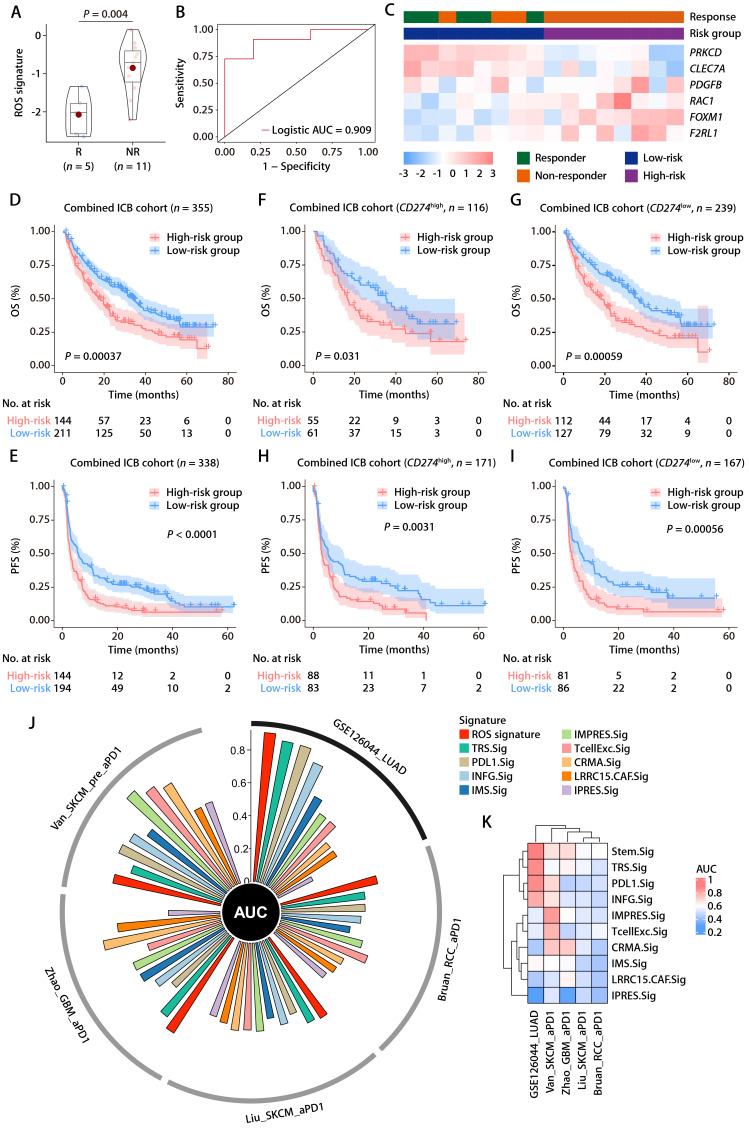
** The predictive value of the ROS-related signature on immunotherapy outcome. (A)** The distributions of the risk scores between responder and non-responder to anti-PD-1 therapy in the GSE126044 dataset. **(B)** Logistic ROC analysis of the ROS-related signature in stratifying responder and non-responder to anti-PD-1 therapy in the GSE126044 dataset. **(C)** The expression pattern of the ROS-related gene signature and response to anti-PD-1 therapy in the GSE126044 dataset.** (D and E)** K-M survival analysis of OS (D) and PFS (E) between the high- and low-risk groups in combined immunotherapy cohorts. **(F and G)** K-M survival analysis of OS between the high- and low-risk groups of patients with high (F) and low (G) *CD274* mRNA expression in combined immunotherapy cohorts. **(H and I)** K-M survival analysis of PFS between the high- and low-risk groups of patients with high (H) and low (I) *CD274* mRNA expression in combined immunotherapy cohorts. **(J)** Circos plot depicting the performance of the ROS-related signature and nine well-established predictive gene signatures of immunotherapy response in the GSE126044_NSCLC_anti-PD-1, Bruan_RCC_aPD1, Liu_SKCM_aPD1, Zhao_GBM_aPD1, and Van_SKCM_pre_aPD1 cohorts. (K) Heatmap comparing the predictive value of the ROS-related signature and nine well-established predictive gene signatures in the GSE126044_NSCLC_anti-PD-1, Bruan_RCC_aPD1, Liu_SKCM_aPD1, Zhao_GBM_aPD1, and Van_SKCM_pre_aPD1 cohorts.

Within the GSE126044 immunotherapy cohort for NSCLC, we computed risk scores employing the identical formula and observed a marked distinction between individuals who responded to anti-PD-1 therapy and those who did not (Fig. 8A). The signature's area under the curve (AUC), serving as a measure for predicting responsiveness to anti-PD-1 treatment, was recorded at 0.909 (Fig. 8B). In addition, the expression patterns of the 6 ROS-related genes were closely related to the immunotherapy response, with *PRKCD* and *CLEC7A* exhibiting positive correlations with and *PDFGB*, *RAC1*, *FOXM1*, and *F2RL1* exhibiting negative correlations with the objective response status (Fig. 8C). Furthermore, the ROS-related signature was extended to the SKCM anti-PD-1 cohort 28 and the UC anti-PD-L1 cohort 29. The results also revealed that the signature was closely related to immunotherapy response and patient outcome (Fig. S5A-S5F). These results indicate that the ROS-related signature can effectively predict the efficacy of immunotherapy. We subsequently combined cohorts of patients treated with anti-PD-1 with RNA-seq data and OS and PFS available, namely, Bruan_RCC_aPD1 (*n* = 181) 30, Liu_SKCM_aPD1 (*n* = 121) 31, Van_SKCM_aPD1 (*n* = 36) 28, and Zhao_GBM_aPD1 (*n* = 17) 32. After data normalization, we scored each patient with the ROS-related signature and classified them into high- and low-risk groups based on the optimal cut-off value. K-M analysis revealed that patients in the high-risk group had worse OS and PFS than those in the low-risk group in the combined immunotherapy cohort (Fig. 8D and 8E). Moreover, higher ROS-related signature expression was associated with significantly worse OS (Fig. 8F and 8G) and PFS (Fig. 8H and 8I) regardless of the *CD274* expression. These data suggested that the ROS-related signature is independent of the PD-L1 expression in predicting the prognosis of patients treated with immunotherapy. Indeed, the ROS-related signature did not significantly correlate with the CD274 mRNA expression (encoding PD-L1) in TCGA-LUAD cohort (Fig. S6A). The *CD274* mRNA expression exhibited no comparable difference in patients with high and low ROS-related signature expression (Fig. S6B). Correlation analysis of TCGA pan-cancer cohorts revealed that the relationship between the ROS-related signature and the *CD274* mRNA expression exhibited heterogeneous patterns (Fig. S6C), in which the ROS-related signature did not correlate significantly with the *CD274* mRNA expression in liver cancer (LIHC), breast cancer (BRCA), esophageal cancer (ESCA), stomach cancer (STAD), prostate cancer (PRAD), endometrioid cancer (UCEC), MESO, rectal cancer (READ), PAAD, ocular melanoma (UVM), uterine carcinosarcoma (UCS), kidney chromophobe (KICH), ACC, and bile duct cancer (CHOL) cohorts (Fig. S6C). Similar results were obtained in the LUAD anti-PD-1 cohort (Fig. S6D and S6E), SKCM anti-PD-1 cohort (Fig. S6F and S6G), and UC anti-PD-L1 cohort (Fig. S6H and S6I), implying that the ROS-related signature was independent of the PD-L1 expression in predicting immunotherapy response and patient outcome. Analysis of the Chinese LUAD cohort also revealed no significant correlation between the ROS-related signature and PD-L1 expression at mRNA and protein levels (Fig. S6J and S6K), further supporting our hypothesis that the ROS-related signature may affect the antitumor immunity and the response to immunotherapy in LUAD through mechanisms beyond the PD-1/PD-L1 axis. Notably, significant correlations were observed between the ROS-related signature and HLA genes in TGCT, GBM, LUSC, SKCM, SARC, OV, HNSC, CESC, PAAD, KIRP, MESO, THCA, COAD, and READ cohorts (Fig. S7), which were similar with the results found in the training and validation cohorts, indicating that the predictive value of ROS-related signature could be extended to other types of solid tumor in addition to LUAD.

We further compared the performance of the ROS-related signature with nine previous well-established predictive gene signatures in LUAD and other types of solid tumors, namely, renal cell carcinoma (RCC), SKCM, and GBM. Compared with TRS.Sig 33, PDL1.Sig 34, INFG.Sig 35, IMS.Sig 36, IMPRES.Sig 37, TcellExc.Sig 38, CRMA.Sig 39, LRRC15.CAF.Sig 40, and IPRES.Sig 41, the ROS-related signature showed the best predictive capability in the GSE126044 NSCLC cohort with an AUC of 0.91 (Fig. 8J) and achieved favorable performance in RCC, SKCM, and GBM cohorts (Fig. 8K), further demonstrating its potential as a predictive model of immune checkpoint blockade (ICB) response in LUAD and other types solid tumors.

Concerning the relationship between the ROS-related signature and the primary/acquired resistance to immunotherapy, we analyzed the GSE91016 dataset in which melanoma patients were treated with anti-PD-1 and anti-CTLA4 therapy 42, and the RNA-seq data of pre- and on-treatment of tissue samples were available, enabling us to investigate the changes of the ROS-related signature upon immunotherapy. K-M survival analysis indicated that OS was significantly lower in the group with high risk compared to the group with low risk based on the ROS-related signature expression of pre-treatment samples of patients treated with immunotherapy (Fig. S8A). However, we did not observe comparable changes in the ROS-related signature expression upon immunotherapy (Fig. S8B and S8C), regardless of the response to immunotherapy (Fig. S8C to S8E). These results indicate that the ROS-related signature was associated with the primary resistance to ICB.

### Translational relevance of the ROS-related signature

**Figure 9:**
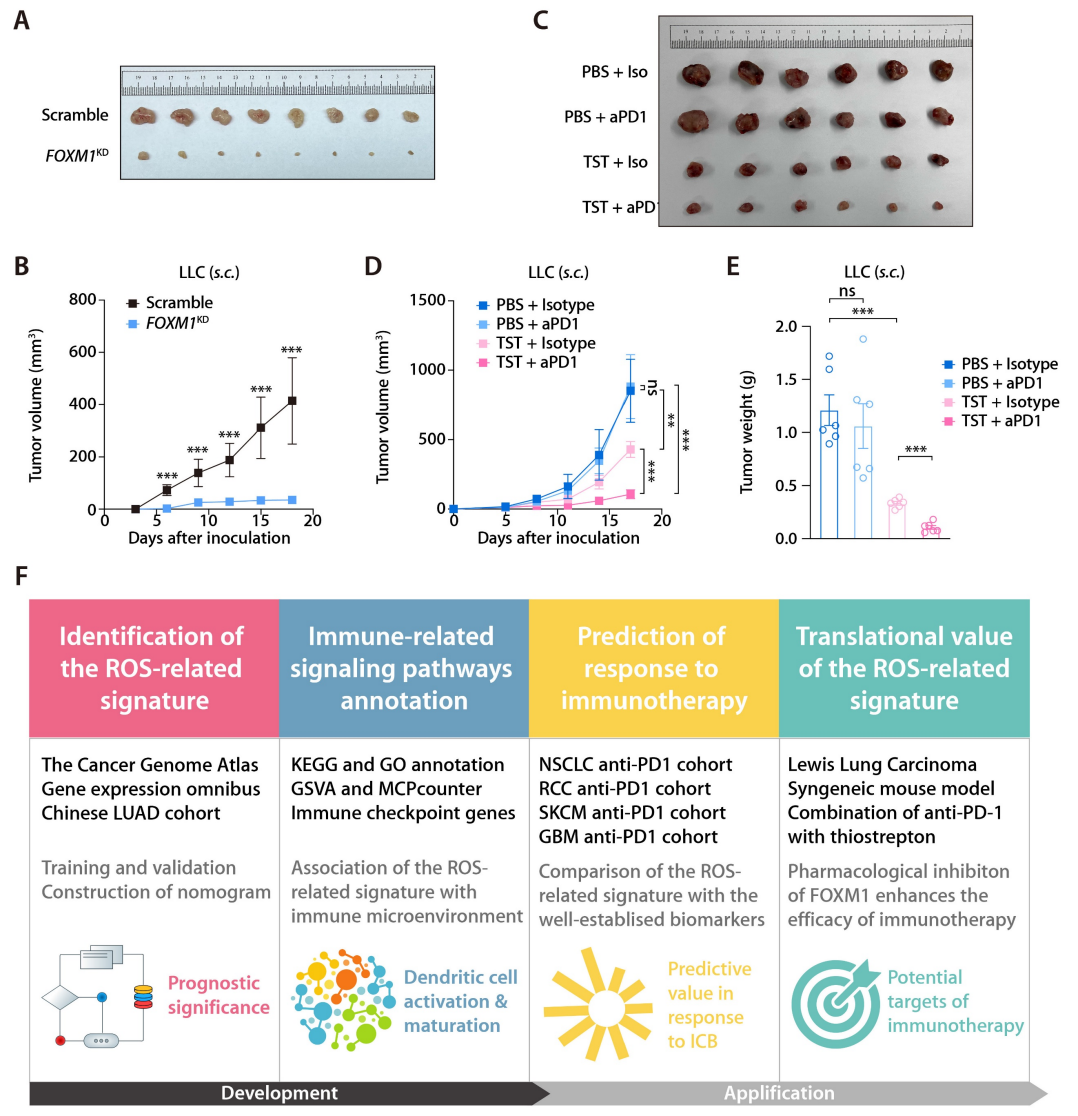
** Inhibition of FOXM1 facilitates response to anti-PD-1 in LLC mouse model. (A)** Subcutaneous C57 mouse xenograft assay of LLC cells transfected with *FOXM1* shRNA (*FOXM1*^KD^) or the scramble control (Scramble) (*n* = 6). Images were acquired on day 17 after inoculation. **(B)** LLC tumor volumes of the *FOXM1*^KD^ and Scramble group as described in (A). **(C)** The representative image of LLC tumors receiving PBS plus Isotype, TST plus isotype, PBS plus anti-PD-1, and TST plus anti-PD-1. **(D)** LLC tumor volumes of different treatment groups (*n* = 6/group) as described in (C). **(E)** LLC tumor weights of different treatment groups (*n* = 6/group) as described in (C). **(F)** Graphical abstract depicting the development of the ROS-related signature and its translational potential.

In the training and validation cohorts, *FOXM1* exhibited the most significant positive correlation with the ROS-related signature, which led us to evaluate the translational relevance of the signature by therapeutically targeting FOXM1. Using short-hairpin RNA-mediated endogenous knockdown of *FOXM1* in LLC tumor cells, we revealed that ablation of tumor-intrinsic FOXM1 attenuated the tumorigenesis capacity of LLC cells *in vivo* (Fig. 9A and 9B). By administrating thiostrepton (TST), which is a protein translation inhibitor that has been reported to inhibit FOXM1 activity 43, we observed that pharmacological inhibition of FOXM1 was sufficient to induce tumor-inhibitory effect and potentiated the response to anti-PD-1 in the LLC mouse model (Fig. 9C to 9E). Taken together, our study has demonstrated the predictive value of the ROS-related signature in LUAD and provided insights into therapeutic targeting of FOXM1 as a novel strategy to potentiate the efficacy of immunotherapy (Fig. 9F).

## Discussion

While considerable research has delved into the interplay between ROS and antitumor immunity, direct clinical evidence linking ROS to immunotherapy response and survival outcome still needs to be improved. Moreover, despite the myriad of studies, biomarkers that account for the complex interplay of intrinsic and extrinsic factors in tumorigenesis for predicting immunotherapy outcomes in LUAD still need to be identified. Hence, there's a pressing demand for novel molecular targets and prognostic indicators to refine LUAD diagnostics and therapeutics. In this context, our study introduces a signature composed of six ROS-associated genes, which we integrated with the TNM staging system to construct a predictive nomogram for OS in LUAD patients. Our analyses underscore the ROS-related signature's excellent capability to differentiate patient outcomes. The nomogram's calibration curve further attests to its accuracy, reflecting a high concordance with actual patient survival rates. Additionally, we propose that the disruption of DC maturation and activation, as revealed through functional annotation and immune infiltration assessments, could contribute to unfavorable prognoses in LUAD patients.

Previous research efforts have been dedicated to identifying molecular markers predicting the prognosis and immunotherapy responses of LUAD patients 44. Such efforts have yielded several credible markers. Specifically, PD-L1 expression, assessed through the IHC method, has been endorsed as a biomarker for immunotherapy response in various solid tumors 45. However, its direct prognostic relevance for patient outcomes remains unclear, and its predictive specificity has yet to meet clinical expectations. Given the sustained survival benefit observed, PD-1/PD-L1 axis blockade has become a preferred first-line therapy for LUAD patients 46. Although immunotherapy has extended survival in clinical settings, the response rate among unselected LUAD patients was approximately 20% due to primary and acquired resistance 47, 48. Most previous studies have concentrated on the immune cell presence or tumor mutational burden on immunotherapy efficacy. Yet, challenges persist, including a lack of transcriptional evidence 49 to support the mechanisms of immunotherapy response and resistance 23. Therefore, the ROS-related signature comprising *FOXM1*, *F2RL1*, *PRKCD*, *CLEC7A*, *PDGFB*, and *RAC1*, emerges as a clinically valuable tool to predict prognosis in patients with LUAD.

ROS function as both tumorigenic agents and inhibitors of cell proliferation, with their effects varying based on the specific spatial and temporal context 50. In the early stages of cancer development, ROS facilitate the onset of cancer by inducing oxidative stress and causing mutations in both pro-oncogenes and genes that suppress tumors. As tumors evolve, ROS contribute to cancer cell invasion and spread by stimulating the NF-κB and MAPK signaling pathways 51. Conversely, at very advanced disease stages, an excess of ROS can halt the cell cycle and trigger apoptosis in malignant cells. ROS initiate extrinsic apoptosis via death receptors and intrinsic apoptosis via mitochondrial routes 51. Additionally, ROS increase levels of beclin-1, an essential autophagy initiator 52, and participate in a critical step of necroptosis 53. These processes have been associated with LUAD development. Prior research indicates that ROS—molecules rich in oxygen that can damage cells in high concentrations—play a critical role in the emergence and progression of LUAD 51. Normally, cells strike a delicate balance between ROS production and elimination. Yet, certain conditions such as persistent inflammation, environmental toxin exposure, or cellular stress can upset this equilibrium, leading to an overabundance of ROS.

In the context of LUAD, various mechanisms contribute to the generation of ROS. A key factor in this process is the enhanced function of NADPH oxidase (NOX) enzymes, which are instrumental in ROS synthesis 54. Elevated NOX activity in LUAD cells is a primary cause of heightened ROS levels. Additionally, mitochondrial dysfunction and the activation of other enzymes, such as xanthine oxidase and cytochrome P450, can lead to ROS production 55. Furthermore, ROS can stimulate transcription factors like NF-κβ, AP-1, and HIF-1α, which are pivotal in gene expression regulation related to these pathways 56. Activation of receptor tyrosine kinases, along with various signaling entities, by ROS can further promote cell growth and survival 57. Considering ROS's crucial role in LUAD, strategies that target ROS or ROS-affected pathways are gaining traction as potential treatments. The use of antioxidants to neutralize ROS and diminish oxidative stress has been explored as a supplementary approach in treating lung cancer. The exploration of inhibitors that specifically target NOX or enzymes that generate ROS is underway, offering promise as potential treatments 58.

Considering the pivotal influence of ROS in LUAD and the therapeutic potential of ROS-related genes, we conducted an in-depth analysis of 235 ROS-related genes in LUAD patient samples from TCGA database. Our findings revealed a dichotomy where a significant portion of these genes acted as protective agents while the remainder posed deleterious effects, echoing the dualistic nature of ROS in the genesis and advancement of tumors. Six genes—*FOXM1*, *F2RL1*, *PRKCD*, *CLEC7A*, *PDGFB*, and *RAC1*— were pinpointed as critical components of the predictive signature. Specifically, *FOXM1*'s upregulation and amplification are implicated in various cancers, including NSCLC, contributing to processes such as invasion, movement, new blood vessel formation, cellular differentiation, and resistance to treatments 59. Past research has indicated that the phosphorylation of FOXM1 at Ser25 can activate genes like IL1A/B, VEGFA, and IL6, which in turn can attract monocytes and promote the differentiation of tumor-associated macrophages (TAMs) towards an M2-like phenotype, thereby aiding in immune escape and fostering an immunosuppressive TME 60. Recent research has linked F2RL1 with poorer outcomes following ICB, T-cell dysfunction in ICB-naive patients and demonstrated resistance to combined PD-1/CTLA-4 blockade in experimental studies 61. F2RL1 encodes PAR2, a G-protein-coupled receptor known to downregulate type I IFN responses and STAT1 signaling 62, leading to the polarization of macrophages towards an M2-like, tumor-promoting phenotype 63. Targeted inhibition of PAR2 has been observed to enhance immunotherapy responsiveness by inducing a phenotypic switch in macrophages 64. These findings position F2RL1 as a promising new target for therapeutic intervention to counteract resistance to immunotherapy. PKCδ, encoded by *PRKCD*, is a cytoplasmic enzyme within the diacylglycerol-responsive and calcium-independent serine/threonine PKC family and acts as a regulatory molecule for apoptosis, being responsive to a spectrum of cellular stress factors, including ultraviolet light exposure, DNA-damaging agents, ROS, growth-promoting signals, and cytokines 64. It influences cellular behaviors like survival, division, movement, and programmed cell death 65. Additionally, this kinase has a widespread presence and fulfills various roles within the cellular components of innate and adaptive immunity 65. PKC-δ is known to govern the migration of neutrophils and their capacity for an oxidative burst 66, 67, and it contributes to the functional capacities of macrophages 68, proliferation and cytokine synthesis in T cells, and B cell receptor signaling pathways 69. The gene *CLEC7A*, which encodes Dectin-1—a member of the C-type lectin receptor family—is predominantly expressed in myeloid-derived suppressor cells (MDSCs) and serves as a receptor for β-1,3-linked glucans 70. Dectin-1's activation orchestrates a spectrum of cellular responses, including the engulfment and digestion of pathogens (phagocytosis), casting of neutrophil extracellular traps, programmed cell death (autophagy), maturation of dendritic cells, presentation of antigens, and the triggering of inflammasomes, including NLRP3 and noncanonical caspase-8 types, as well as the release of eicosanoids and signaling proteins like cytokines and chemokines 71. Recent research has shown that Dectin-1 signaling can prompt the release of pro-allergic chemokines and the secretion of mucus 72, drive the differentiation of regulatory T cells 73, and activate IL-17F signaling pathways 74, 75, suggesting that Dectin-1 has critical roles in not only the immune response but also in the development of allergic, immune-mediated, and neoplastic diseases 76. PDGFB is implicated in various fundamental biological processes like embryonic development, tissue repair, and the healing of wounds. However, irregularities in the signaling pathway of PDGFB are linked to the onset and advancement of cancer by fostering cell proliferation, stimulating the formation of new blood vessels, and increasing the invasive potential of cancerous cells 77. Consequently, PDGFB has emerged as a potential focal point for therapeutic strategies. Currently available targeted therapies that act on PDGFB gene fusions have demonstrated notable efficacy in treating patients with these specific gene fusions 77. In the context of PDGFB-driven GBM, these cells exhibit a distinct TME phenotype, where PDGFB modulates the secretion of monocyte chemoattractant proteins, leading to the recruitment of bone marrow-derived macrophages and the production of IL-1β 78. Interrupting the IL-1β/IL1R1 inflammatory cytokine feedback loop has improved survival rates and reduced the prevalence of IBA1-positive tumor-associated macrophages within PDGFB-driven GBM 78. Additionally, recent research indicates that PDGFB-based nanocomposites may enhance the movement and infiltration of natural killer cells, macrophages that exhibit an M1 phenotype, and CD8^+^ T cells, thereby strengthening antitumor immune response and curtailing tumor growth. These findings offer a glimpse into a potential new approach for the immunotherapy of solid tumors 79. RAC1, a small GTPase from the Rac subfamily, is instrumental in various cellular processes, including cell migration. Abnormal RAC1 activity contributes to irregular cell movement, making it a significant molecule in cancer research. This is particularly due to its overexpression in various aggressive tumors, positioning RAC1 as a pivotal target for cancer treatment strategies 80. Activation of RAC1 is known to regulate B-cell-mediated humoral immunity and *in vitro* immunoglobulin class switching 81, mediate harmful interactions between epithelial cells and immune cells 82, and maintain immune homeostasis 83. Moreover, RAC1, along with TIAM1, is implicated in interleukin 17A (IL-17A) transcription and has a role in autoimmunity 84. Dysregulated RAC1 activity has been linked to alterations in IL-10 mediated signaling pathways and inflammatory cytokine levels 85, as well as the preservation of T cell adhesion and motility and the regulation of LFA-1 integrin functions 86, which is critical for lymphocyte activation. Collectively, these insights underscore the essential functions of the six genes comprising the ROS-related signature in the modulation of adaptive immunity and antitumor responses and affirm the ROS-related signature's utility in predicting patient outcomes, TME phenotype, and the response to ICB.

In an examination of LUAD at the transcriptome level, a consistent pattern of diminished expression of genes tied to immune function and a lower presence of immune cells within tumors expressing a high ROS-related signature was noted. Intriguingly, an inverse correlation was found between the expression of HLA genes and the ROS-related signature. Additionally, the potential impact of DCs on ICI response through the tertiary lymphoid structure (TLS) 87 prompted an analysis of DCs' correlation with the ROS-related signature, which showed a negative relationship for both immature and activated DCs. Subsequent investigations also highlighted a significant link between the ROS-related signature and various immune-related biological processes, such as the adaptive immune response, antigen processing and presentation, MHC complex binding, migration of myeloid leukocytes, and lymphocyte-driven immunity. Furthermore, tumors exhibiting a high ROS-related signature were posited to have abnormal activations of pathways associated with hypoxia, Wnt/β-catenin, and TNF-α signaling via NF-κβ. The development of a hypoxic phenotype is recognized as a progressive tactic by which tumors circumvent immune detection 88. Moreover, the intrinsic activation of Wnt/β-catenin signaling has been pinpointed as a critical factor in creating a T-cell-excluded TME 89. The association of high ROS-related signature expression with pronounced immunosuppressive characteristics supports the predictive potential of this signature.

The efficacy of the ROS-related signature in prognostication was corroborated across multiple independent cohorts and distinct clinical subdivisions, prompting further investigation into its mechanism for predicting outcomes. Functional annotation of DEGs between groups stratified by ROS-related risk levels underscored a significant engagement in immune-centric biological processes and pathways, including antigen processing and presentation, and MHC complex interactions. This suggests that immunological diversity may be a crucial element influencing OS disparities between high- and low-risk categories. Further assessments involving five immune-related clusters and scrutiny of immune cell infiltration furnished a deeper understanding of the immune milieu's variation between these groups. It was established that patients with a higher risk profile were characterized by an immunosuppressed status, marked by scant tumor-infiltrating leukocytes, particularly DCs, which are pivotal for antigen presentation. Conversely, low-risk patients demonstrated robust infiltration of activated CD8^+^ T cells and M1 macrophages, indicating a more vigorous antitumor immune response. Indeed, IHC analysis of tissue samples from the Chinese LUAD cohort demonstrated a negative correlation between the ROS-related signature and infiltration of DCs and CD8^+^ T cells. Nonetheless, the impact of these underlying mechanisms of the ROS-related signature on DC maturation and activation warrants subsequent experimental exploration.

While the ROS-related signature shows promise as a reliable, independent predictive tool and a potential predictor of immunotherapy responses in LUAD patients, certain limitations must be considered. Firstly, our study relied on retrospective data, and there is a need for validation with prospective samples to confirm these findings. Secondly, the selection of candidate genes was limited to those responding to ROS. Given the high spatial heterogeneity of the tumor immune microenvironment (TIME), this may have restricted the prognostic predictive capacity of the ROS-related signature. Nevertheless, the signature contributes valuable insights into the immune microenvironment and potential immunotherapy responses. Thirdly, this study did not directly assess patients undergoing immunotherapy, meaning that the signature's predictive accuracy for immunotherapy response was inferred rather than directly observed. Consequently, there is a clear need for future prospective studies with sufficient power to further elucidate the signature's utility in clinical settings. Because tissue-based transcriptomic data from on- or post-treatment settings are relatively difficult to obtain 23, the relevance of the ROS-related signature with the acquired resistance to ICB warrants further exploration.

## Conclusion

ROS play a pivotal role in the tumorigenesis and progression of LUAD. Our research delved into the expression and clinical relevance of genes associated with ROS in LUAD patients. We developed a predictive model based on these ROS-related genes, rigorously validating its efficacy across various LUAD patient cohorts and immunotherapy cohorts. These insights have implications for refining clinical assessments and optimizing immunotherapeutic strategies for patients with LUAD and other types of solid tumors.

## Supplementary Material

Supplementary figures.

Supplementary tables.
