# Supplementary material for: Discovery of a novel ROS-based signature for predicting prognosis and immunosuppressive tumor microenvironment in lung adenocarcinoma
Source: J Cancer. 2024 Mar 17;15(9):2691–711. doi: 10.7150/jca.93975 (PMC10988302; doi:10.7150/jca.93975)
Supplement: Supplementary file 1 — Supplementary figures. [file jcav15p2691s1.pdf]

A

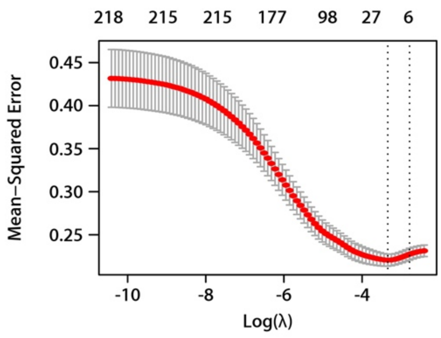

218 215 215 177 98 27 6
0.45
0.40
0.35
0.30
0.25
Mean−Squared Error
-10 -8 -6 -4
Log(λ)

B

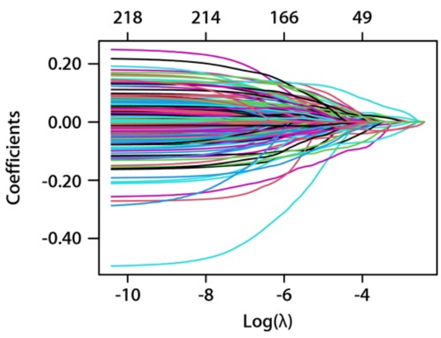

218 214 166 49
0.20
0.00
-0.20
-0.40
Coefficients
-10 -8 -6 -4
Log(λ)

Fig. S1. LASSO regression analysis of the ROS-related genes.

(A) LASSO regression of the 235 ROS-related genes. (B) Cross-validation for tuning the parameter selection in the LASSO regression.

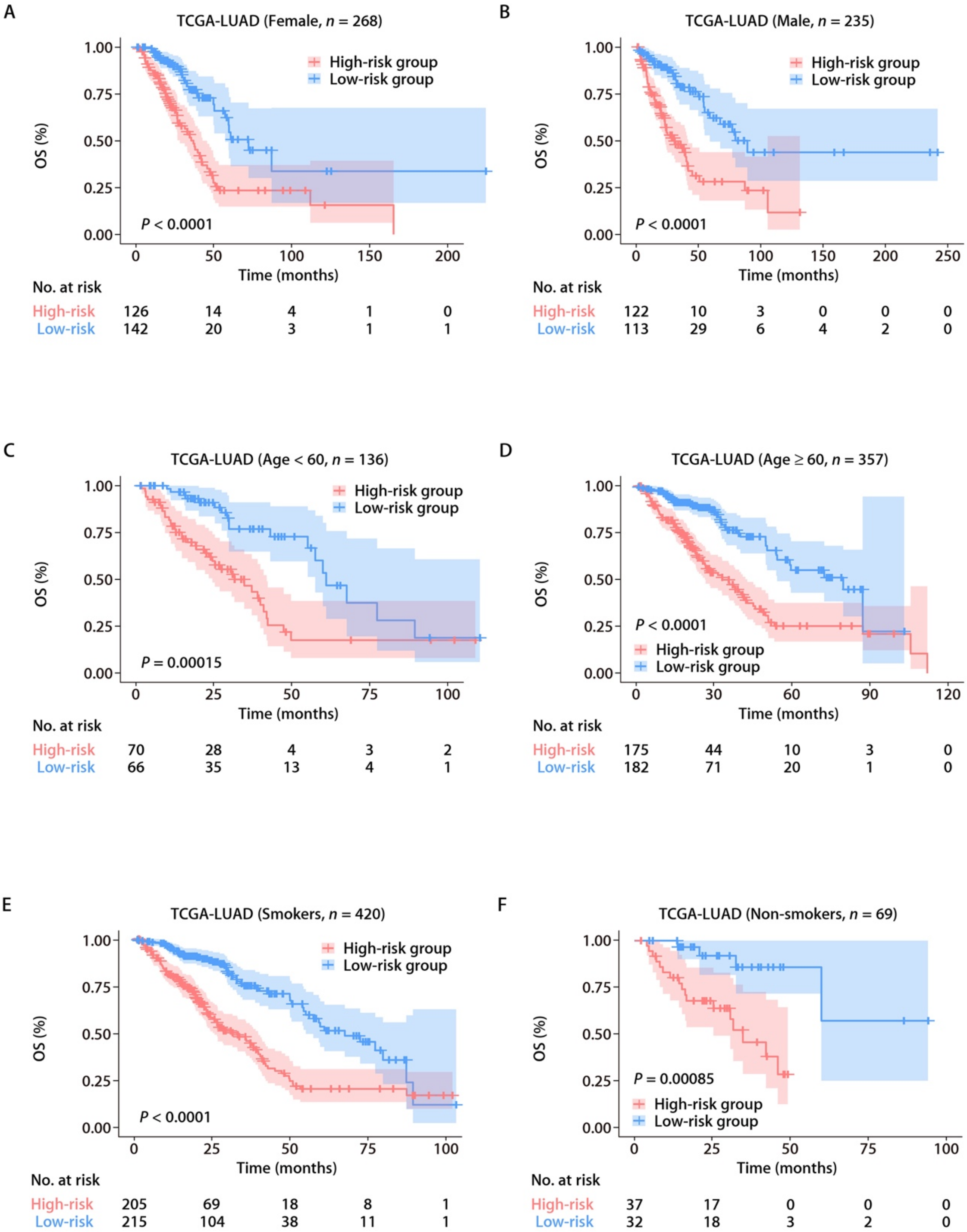
A
TCGA-LUAD (Female, n = 268)
High-risk group
Low-risk group
P < 0.0001
OS (%)
1.00
0.75
0.50
0.25
0.00
0
50
100
150
200
Time (months)
No. at risk
High-risk 126 14 4 1 0
Low-risk 142 20 3 1 1
B
TCGA-LUAD (Male, n = 235)
High-risk group
Low-risk group
P < 0.0001
OS (%)
1.00
0.75
0.50
0.25
0.00
0
50
100
150
200
250
Time (months)
No. at risk
High-risk 122 10 3 0 0 0
Low-risk 113 29 6 4 2 0
C
TCGA-LUAD (Age < 60, n = 136)
High-risk group
Low-risk group
P = 0.00015
OS (%)
1.00
0.75
0.50
0.25
0.00
0
25
50
75
100
Time (months)
No. at risk
High-risk 70 28 4 3 2
Low-risk 66 35 13 4 1
D
TCGA-LUAD (Age ≥ 60, n = 357)
P < 0.0001
High-risk group
Low-risk group
OS (%)
1.00
0.75
0.50
0.25
0.00
0
30
60
90
120
Time (months)
No. at risk
High-risk 175 44 10 3 0
Low-risk 182 71 20 1 0
E
TCGA-LUAD (Smokers, n = 420)
High-risk group
Low-risk group
P < 0.0001
OS (%)
1.00
0.75
0.50
0.25
0.00
0
25
50
75
100
Time (months)
No. at risk
High-risk 205 69 18 8 1
Low-risk 215 104 38 11 1
F
TCGA-LUAD (Non-smokers, n = 69)
P = 0.00085
High-risk group
Low-risk group
OS (%)
1.00
0.75
0.50
0.25
0.00
0
25
50
75
100
Time (months)
No. at risk
High-risk 37 17 0 0 0
Low-risk 32 18 3 2 0

Fig. S2. Validation of the ROS-related signature in clinical subgroups.

K-M curves of OS in female (A), male (B), younger (C), older (D), smokers (E), and non-smokers (F) patients based on risk scores in TCGA-LUAD cohort.

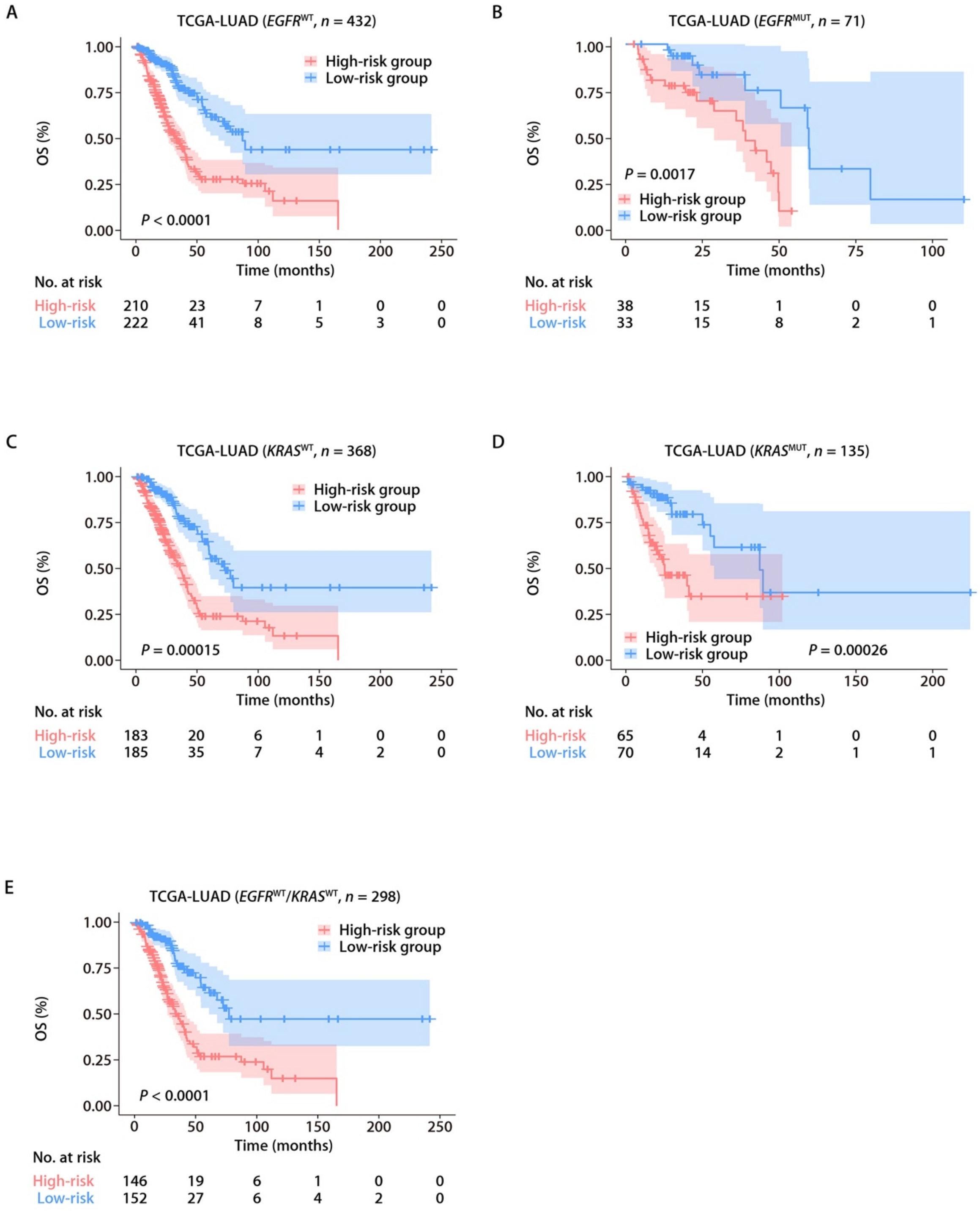
A
TCGA-LUAD (EGFR^WT, n = 432)
High-risk group
Low-risk group
P < 0.0001
OS (%)
Time (months)
No. at risk
High-risk 210 23 7 1 0 0
Low-risk 222 41 8 5 3 0
B
TCGA-LUAD (EGFR^MUT, n = 71)
P = 0.0017
High-risk group
Low-risk group
OS (%)
Time (months)
No. at risk
High-risk 38 15 1 0 0
Low-risk 33 15 8 2 1
C
TCGA-LUAD (KRAS^WT, n = 368)
High-risk group
Low-risk group
P = 0.00015
OS (%)
Time (months)
No. at risk
High-risk 183 20 6 1 0 0
Low-risk 185 35 7 4 2 0
D
TCGA-LUAD (KRAS^MUT, n = 135)
High-risk group
Low-risk group
P = 0.00026
OS (%)
Time (months)
No. at risk
High-risk 65 4 1 0 0
Low-risk 70 14 2 1 1
E
TCGA-LUAD (EGFR^WT/KRAS^WT, n = 298)
High-risk group
Low-risk group
P < 0.0001
OS (%)
Time (months)
No. at risk
High-risk 146 19 6 1 0 0
Low-risk 152 27 6 6 4 2 0

Fig. S3. Validation of the ROS-related signature in different mutation statuses.

K-M curves were plotted to estimate the OS for patients carrying *EGFR*^{WT} (A), *EGFR*^{MUT} (B), *KRAS*^{WT} (C), *KRAS*^{MUT} (D), and *EGFR*^{WT}/*KRAS*^{WT} (E) based on the risk scores in TCGA-LUAD cohort.

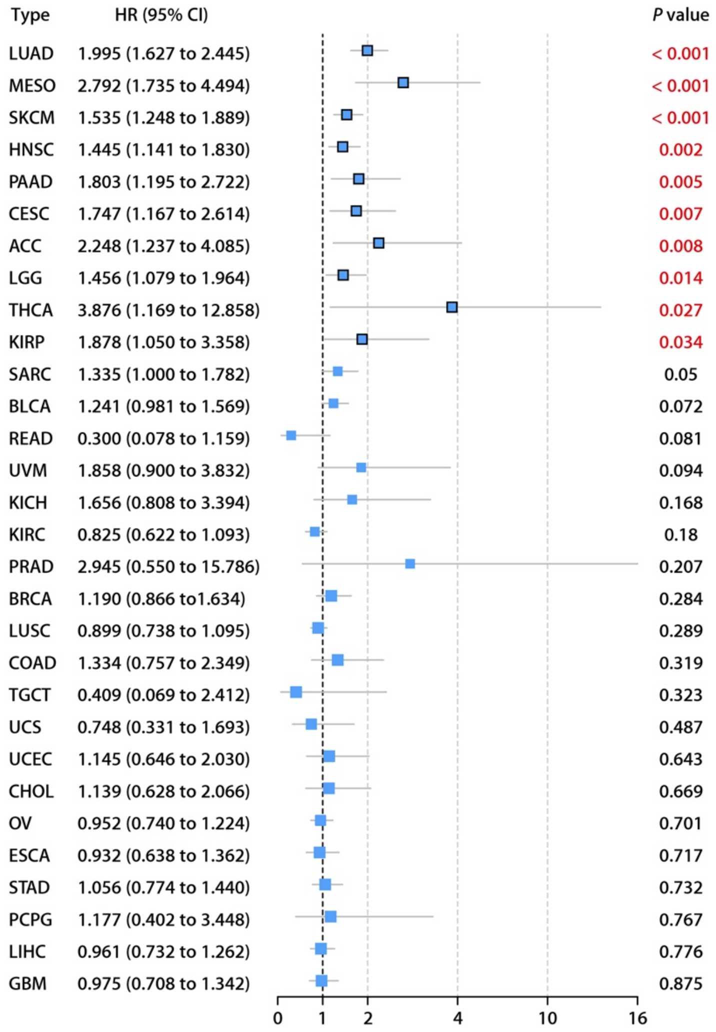
Type
HR (95% CI)
P value
LUAD 1.995 (1.627 to 2.445) < 0.001
MESO 2.792 (1.735 to 4.494) < 0.001
SKCM 1.535 (1.248 to 1.889) < 0.001
HNSC 1.445 (1.141 to 1.830) 0.002
PAAD 1.803 (1.195 to 2.722) 0.005
CESC 1.747 (1.167 to 2.614) 0.007
ACC 2.248 (1.237 to 4.085) 0.008
LGG 1.456 (1.079 to 1.964) 0.014
THCA 3.876 (1.169 to 12.858) 0.027
KIRP 1.878 (1.050 to 3.358) 0.034
SARC 1.335 (1.000 to 1.782) 0.05
BLCA 1.241 (0.981 to 1.569) 0.072
READ 0.300 (0.078 to 1.159) 0.081
UVM 1.858 (0.900 to 3.832) 0.094
KICH 1.656 (0.808 to 3.394) 0.168
KIRC 0.825 (0.622 to 1.093) 0.18
PRAD 2.945 (0.550 to 15.786) 0.207
BRCA 1.190 (0.866 to1.634) 0.284
LUSC 0.899 (0.738 to 1.095) 0.289
COAD 1.334 (0.757 to 2.349) 0.319
TGCT 0.409 (0.069 to 2.412) 0.323
UCS 0.748 (0.331 to 1.693) 0.487
UCEC 1.145 (0.646 to 2.030) 0.643
CHOL 1.139 (0.628 to 2.066) 0.669
OV 0.952 (0.740 to 1.224) 0.701
ESCA 0.932 (0.638 to 1.362) 0.717
STAD 1.056 (0.774 to 1.440) 0.732
PCPG 1.177 (0.402 to 3.448) 0.767
LIHC 0.961 (0.732 to 1.262) 0.776
GBM 0.975 (0.708 to 1.342) 0.875
0
1
2
4
10
16

Fig. S4. Multivariate Cox regression analysis of the ROS-related signature in TCGA pan-cancer cohorts.

Forest plot showing hazard ratio (HR) of the ROS-related signature on the OS in TCGA pan-cancer cohort. Error bars are 95% confidence intervals.

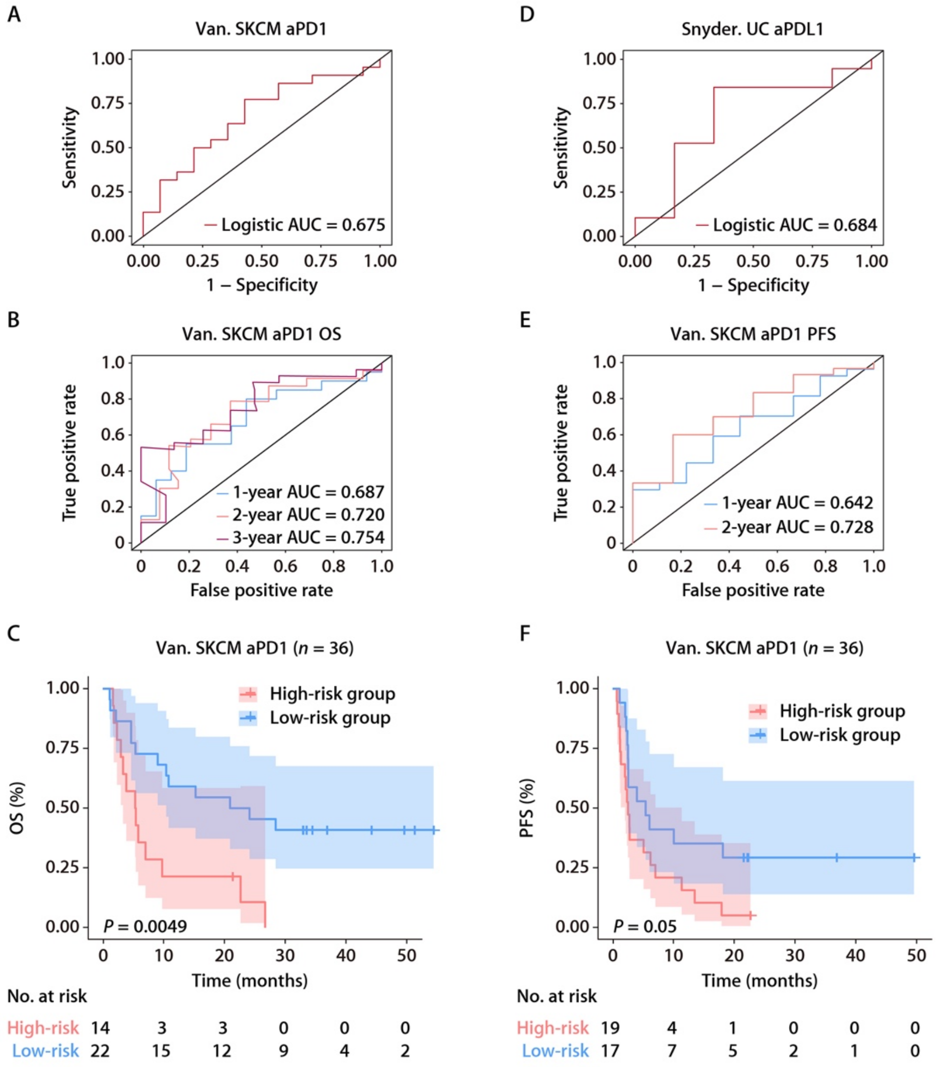
A
Van. SKCM aPD1
Sensitivity
1 – Specificity
Logistic AUC = 0.675
D
Snyder. UC aPDL1
Logistic AUC = 0.684
B
Van. SKCM aPD1 OS
True positive rate
False positive rate
1-year AUC = 0.687
2-year AUC = 0.720
3-year AUC = 0.754
E
Van. SKCM aPD1 PFS
1-year AUC = 0.642
2-year AUC = 0.728
C
Van. SKCM aPD1 (n = 36)
OS (%)
High-risk group
Low-risk group
P = 0.0049
Time (months)
No. at risk
High-risk 14 3 3 0 0 0
Low-risk 22 15 12 9 4 2
F
Van. SKCM aPD1 (n = 36)
PFS (%)
P = 0.05
No. at risk
High-risk 19 4 1 0 0 0
Low-risk 17 7 5 2 1 0

Fig. S5. Prediction of ICI outcome using the ROS-related signature.

(A) ROC plot depicting the performance of the ROS-related signature in the SKCM anti-PD-1 cohort. (B) Time-dependent ROC analysis assessing the predictive function of the ROS-related signature at 1-, 3- and 5-year OS in SKCM anti-PD-1 cohort. (C) K-M survival analysis of OS between the high- and low-risk groups in the SKCM anti-PD-1 cohort. (D) ROC plot depicting the performance of the ROS-related signature in the UC anti-PD-L1 cohort. (E) Time-dependent ROC analysis to assess the predictive function of the ROS-related signature at 1- and 2-year PFS in the SKCM anti-PD-1 cohort. (F) K-M survival analysis of PFS between the high- and low-risk groups in the SKCM anti-PD-1 cohort.

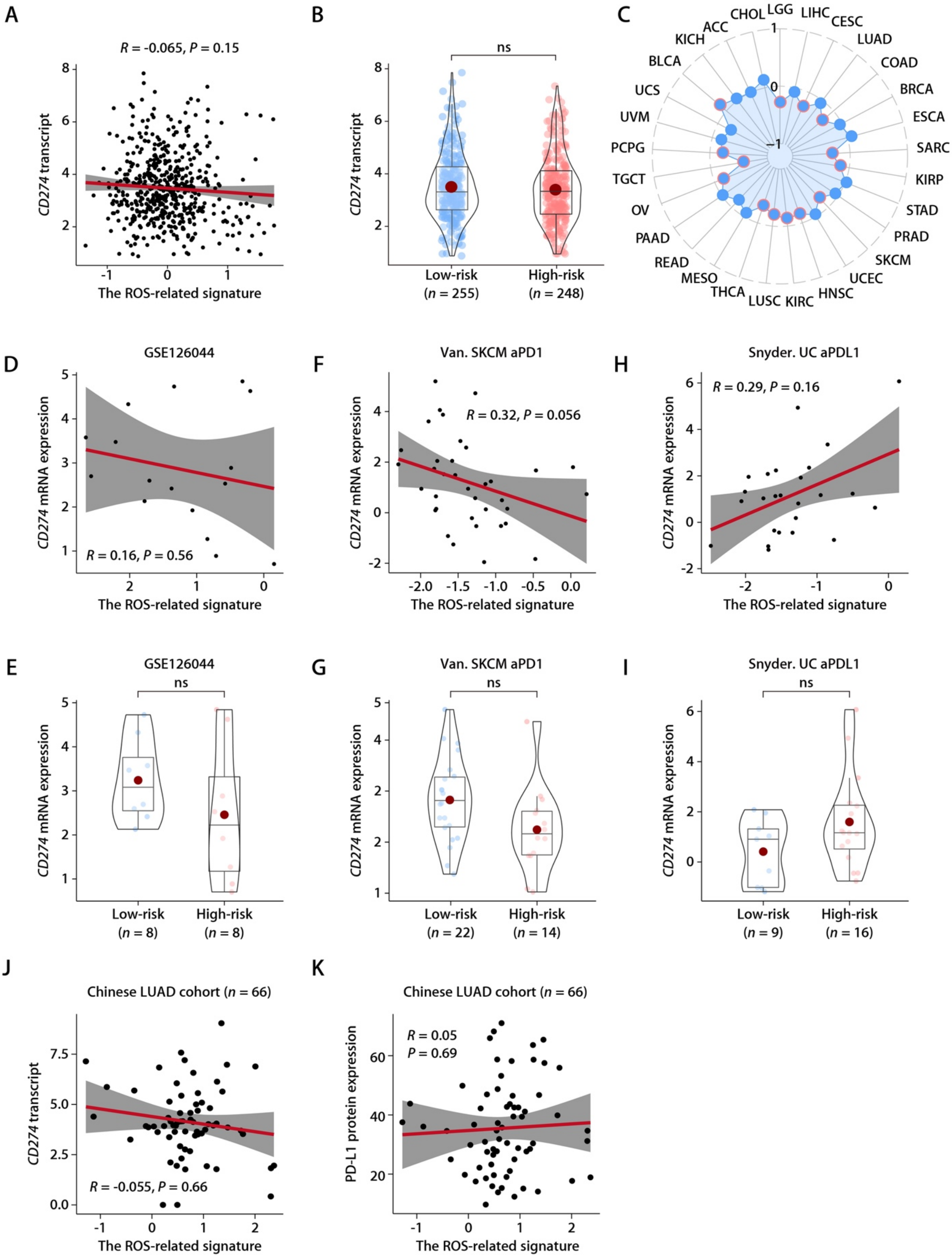

A
R = -0.065, P = 0.15
CD274 transcript
The ROS-related signature
B
ns
CD274 transcript
Low-risk
(n = 255)
High-risk
(n = 248)
C
LGG
LIHC
CESC
LUAD
COAD
BRCA
ESCA
SARC
KIRP
STAD
PRAD
SKCM
UCEC
HNSC
KIRC
LUSC
THCA
MESO
READ
PAAD
OV
TGCT
PCPG
UVM
UCS
BLCA
KICH
ACC
CHOL
D
GSE126044
CD274 mRNA expression
R = 0.16, P = 0.56
The ROS-related signature
F
Van. SKCM aPD1
CD274 mRNA expression
R = 0.32, P = 0.056
The ROS-related signature
H
Snyder. UC aPDL1
CD274 mRNA expression
R = 0.29, P = 0.16
The ROS-related signature
E
GSE126044
ns
CD274 mRNA expression
Low-risk
(n = 8)
High-risk
(n = 8)
G
Van. SKCM aPD1
ns
CD274 mRNA expression
Low-risk
(n = 22)
High-risk
(n = 14)
I
Snyder. UC aPDL1
ns
CD274 mRNA expression
Low-risk
(n = 9)
High-risk
(n = 16)
J
Chinese LUAD cohort (n = 66)
CD274 transcript
R = -0.055, P = 0.66
The ROS-related signature
K
Chinese LUAD cohort (n = 66)
PD-L1 protein expression
R = 0.05
P = 0.69
The ROS-related signature

Fig. S6. Correlation analysis of the ROS-related signature and PD-L1 expression.

(A) Correlation analysis between the ROS-related signature and *CD274* mRNA expression in TCGA-LUAD cohort. (B) The distributions of *CD274* mRNA expression between the high- and low-risk groups in TCGA-LUAD cohort. (C) Circos plot depicting the Pearson correlation between the ROS-related signature and *CD274* mRNA expression in TCGA pan-cancer cohorts. The red circle indicates a significant correlation. (D) Correlation analysis between the ROS-related signature and *CD274* mRNA expression in the GSE126044 NSCLC anti-PD-1 cohort. (E) The distributions of *CD274* mRNA expression between the high- and low-risk groups in the GSE126044 NSCLC anti-PD-1 cohort. (F) Correlation analysis between the ROS-related signature and *CD274* mRNA expression in the SKCM anti-PD-1 cohort. (G) The distributions of *CD274* mRNA expression between the high- and low-risk groups in the SKCM anti-PD-1 cohort. (H) Correlation analysis between the ROS-related signature and *CD274* mRNA expression in the UC anti-PD-L1 cohort. (I) The distributions of *CD274* mRNA expression between the high- and low-risk groups in the UC anti-PD-L1 cohort. (J) Correlation analysis between the ROS-related signature and *CD274* mRNA expression in the Chinese LUAD cohort. (K) Correlation analysis between the ROS-related signature and PD-L1 protein expression quantified by IHC analysis in the Chinese LUAD cohort.

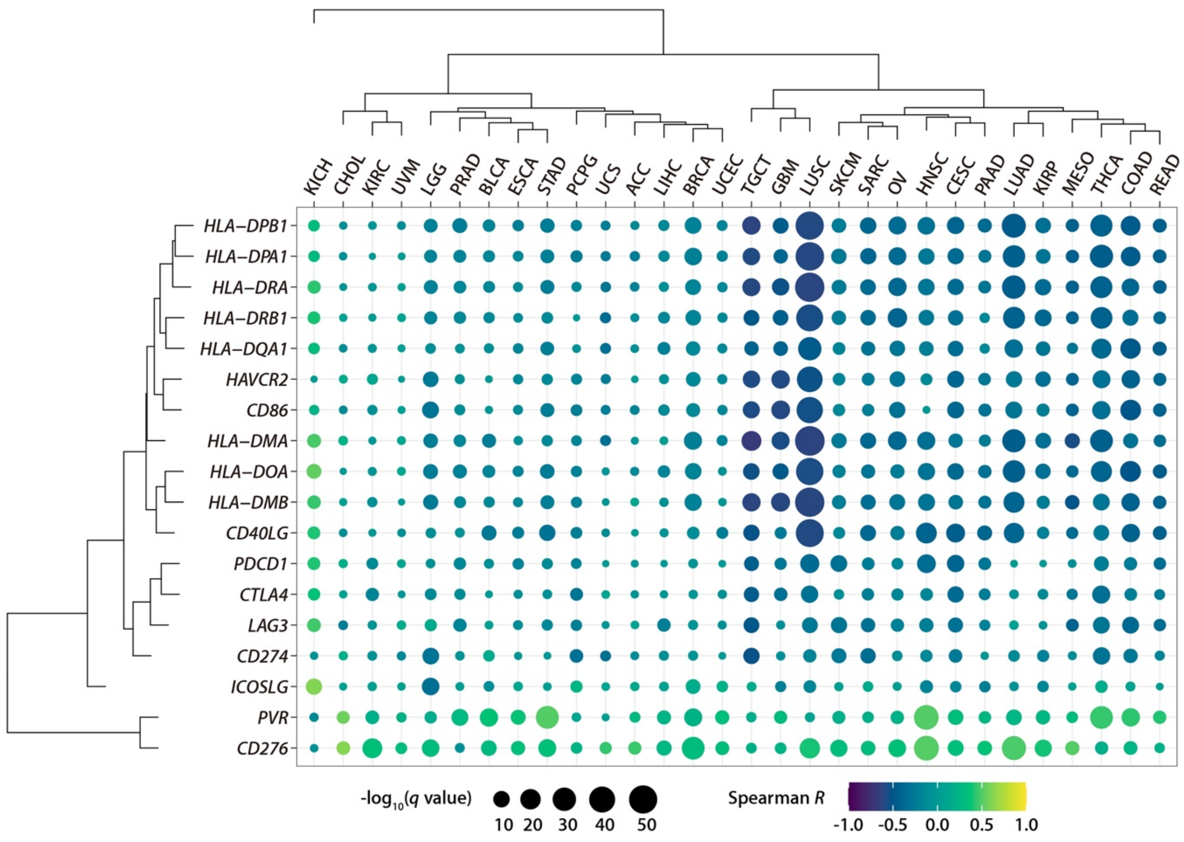

KICH
CHOL
KIRC
UVM
LGG
PRAD
BLCA
ESCA
STAD
PCPG
UCS
ACC
LIHC
BRCA
UCEC
TGCT
GBM
LUSC
SKCM
SARC
OV
HNSC
CESC
PAAD
LUAD
KIRP
MESO
THCA
COAD
READ
HLA–DPB1
HLA–DPA1
HLA–DRA
HLA–DRB1
HLA–DQA1
HAVCR2
CD86
HLA–DMA
HLA–DOA
HLA–DMB
CD40LG
PDCD1
CTLA4
LAG3
CD274
ICOSLG
PVR
CD276
-log10(q value)
10 20 30 40 50
Spearman R
-1.0 -0.5 0.0 0.5 1.0

Fig. S7. Correlation analysis of the ROS-related signature and immune checkpoint genes.

Heatmap depicting the correlation between the ROS-related signature and the expressions of immune checkpoint genes in TCGA-LUAD cohort.

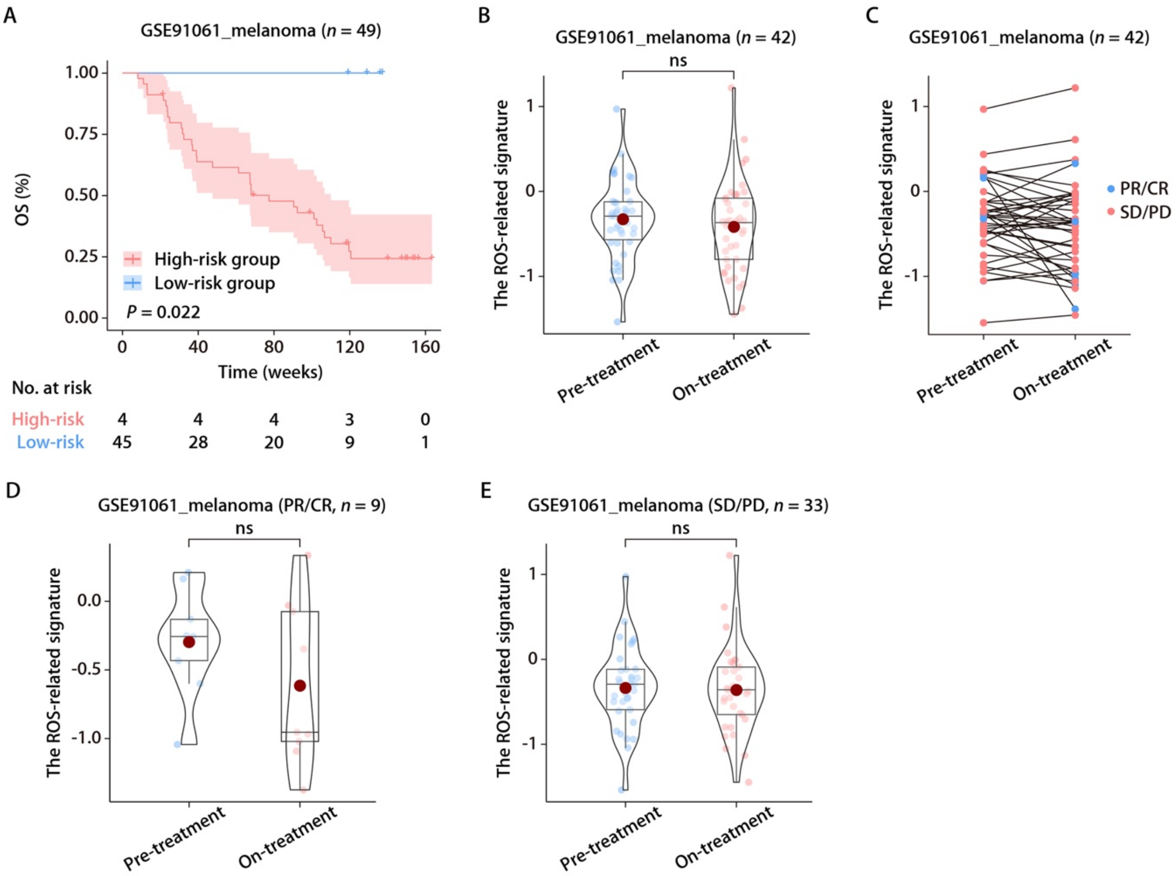
A
GSE91061_melanoma (n = 49)
1.00
0.75
0.50
0.25
0.00
OS (%)
High-risk group
Low-risk group
P = 0.022
0
40
80
120
160
Time (weeks)
No. at risk
High-risk 4 4 4 3 0
Low-risk 45 28 20 9 1
B
GSE91061_melanoma (n = 42)
ns
The ROS-related signature
Pre-treatment
On-treatment
C
GSE91061_melanoma (n = 42)
PR/CR
SD/PD
D
GSE91061_melanoma (PR/CR, n = 9)
E
GSE91061_melanoma (SD/PD, n = 33)

Fig. S8. The association of the ROS-related signature with the primary resistance to immunotherapy.

(A) K-M survival analysis of OS between the high- and low-risk groups stratified by the ROS-related signature in the GSE96061 melanoma immunotherapy cohort. (B) The distributions of the ROS-related signature expression between the pre- and on-treatment samples of patients in the GSE96061 melanoma immunotherapy cohort. (C) Comparison of the ROS-related signature expression between tumor samples of response ($n = 9$) and non-response ($n = 33$) to immunotherapy in the GSE91061 melanoma immunotherapy cohort. (D) The distributions of the ROS-related signature expression between the pre- and on-treatment samples of patients with response to immunotherapy in the GSE96061 melanoma immunotherapy cohort. (E) The distributions of the ROS-related signature expression between the pre- and on-treatment samples of patients with no response to immunotherapy in the GSE96061 melanoma immunotherapy cohort.
