# Supplementary material for: Discovery of a novel ROS-based signature for predicting prognosis and immunosuppressive tumor microenvironment in lung adenocarcinoma
Source: J Cancer. 2024 Mar 17;15(9):2691–711. doi: 10.7150/jca.93975 (PMC10988302; doi:10.7150/jca.93975)
Supplement: Supplementary file 2 — Supplementary tables. [file jcav15p2691s2.zip › final_tables_20240312/Table S6_Univariate Cox regression for OS in training and validation cohorts.docx]

**Table S6. Univariate Cox regression for OS in training and validation cohorts**

| **Characteristics** | **TCGA-LUAD** | | **GSE50081** | | **GSE13213** | | **GSE29016** | | |
| --- | --- | --- | --- | --- | --- | --- | --- | --- | --- |
|  | **HR (95% CI)** | ***P* value** | **HR (95% CI)** | ***P* value** | **HR (95% CI)** | ***P* value** | **HR (95% CI)** | ***P* value** |  |
| Age |  | 0.918 |  | 0.376 |  | 0.863 |  | 0.375 |  |
| < 60 | 1 |  | 1 |  | 1 |  | 1 |  |  |
| ≥ 60 | 1.017 (0.733-1.412) |  | 1.471 (0.626-3.457) |  | 1.054 (0.581-1.912) |  | 1.507 (0.609-3.730) |  |  |
| Gender |  | 0.671 |  | 0.228 |  | 0.348 |  | 0.281 |  |
| Female | 1 |  | 1 |  | 1 |  | 1 |  |  |
| Male | 1.065 (0.795-1.427) |  | 1.410 (0.807-2.463) |  | 1.316 (0.742-2.335) |  | 1.506 (0.715-3.171) |  |  |
| Smoking history |  | 0.823 |  | 0.194 |  | 0.243 |  | 0.652 |  |
| No | 1 |  | 1 |  | 1 |  | 1 |  |  |
| Yes | 0.953 (0.622-1.458) |  | 1.698 (0.763-3.777) |  | 1.409 (0.792-2.506) |  | 1.235 (0.494-3.085) |  |  |
| T stage |  | < 0.001 |  | 0.004 |  | 0.300 |  | 0.100 |  |
| T0 and T1 | 1 |  | 1 |  | 1 |  | 1 |  |  |
| T2 | 1.548 (1.079-2.221) | 0.018 | 2.441 (1.217-4.896) | 0.012 | 1.352 (0.721-2.534) | 0.347 | 0.380 (0.156-0.925) | 0.033 |  |
| T3 | 3.006 (1.764-5.121) | < 0.001 | 11.731 (2.502-54.992) | 0.002 | 1.494 (0.508-4.393) | 0.465 | 0.600 (0.130-2.765) | 0.512 |  |
| T4 | 3.167 (1.626-6.172) | < 0.001 | / |  | 3.112 (1.052-9.209) | 0.040 | 2.609 (0.548-12.421) | 0.228 |  |
| TX | 5.111 (1.226-21.300) | 0.025 | / |  | / |  | 1.647 (0.202-13.410) | 0.641 |  |
| N stage |  | < 0.001 |  | 0.010 |  | 0.002 |  | 0.319 |  |
| N0 | 1 |  | 1 |  | 1 |  | 1 |  |  |
| N1 | 2.482 (1.760-3.500) | < 0.001 | 2.142 (1.199-3.825) |  | 2.016 (0.703-5.777) | 0.192 | / |  |  |
| N2 | 3.099 (2.109-4.554) | < 0.001 | / |  | 3.323 (1.784-6.189) | < 0.001 | / |  |  |
| N3 | Inf | 0.994 | / |  | / |  | / |  |  |
| NX | 1.472 (0.539-4.020) | 0.451 | / |  | / |  | 2.832 (0.365-21.960) |  |  |
| M stage |  | 0.020 |  | / |  | / |  | / |  |
| M0 | 1 |  |  |  |  |  |  |  |  |
| M1 | 2.176 (1.272-3.724) | 0.005 |  |  |  |  |  |  |  |
| MX | 0.855 (0.594-1.229) | 0.398 |  |  |  |  |  |  |  |
| Risk group |  | < 0.001 |  | < 0.001 |  | < 0.001 |  | 0.002 |  |
| Low-risk | 1 |  | 1 |  | 1 |  | 1 |  |  |
| High-risk | 3.010 (2.196-4.125) |  | 2.717 (1.564-4.720) |  | 4.251 (1.901-9.506) |  | 3.503 (1.592-7.708) |  |  |
