# Supplementary material for: Discovery of a novel ROS-based signature for predicting prognosis and immunosuppressive tumor microenvironment in lung adenocarcinoma
Source: J Cancer. 2024 Mar 17;15(9):2691–711. doi: 10.7150/jca.93975 (PMC10988302; doi:10.7150/jca.93975)
Supplement: Supplementary file 2 — Supplementary tables. [file jcav15p2691s2.zip › final_tables_20240312/Table S2_Clinicopathological characteristics of LUAD patients in the training and validation cohorts.docx]

**Table S2. Clinicopathological characteristics of LUAD patients in the training and validation cohorts**

| **Characteristics** | **Training cohort** | **Validation cohort** | | | | | | |
| --- | --- | --- | --- | --- | --- | --- | --- | --- |
|  | **TCGA-LUAD** | **GSE50081** | **GSE13213** | **GSE30219** | **GSE72094** | **GSE29016** | **GSE26939** | **LUAD cohort** |
|  | ***n* = 503** | ***n* = 127** | ***n* = 117** | ***n* = 85** | ***n* = 442** | ***n* = 38** | ***n* = 116** | ***n* = 66** |
| Age |  |  |  |  |  |  |  |  |
| < 60 | 136 | 19 | 43 | 37 | 60 | 10 | 38 | 20 |
| ≥ 60 | 357 | 108 | 74 | 48 | 361 | 28 | 78 | 46 |
| NA | 10 | 0 | 0 | 0 | 21 | 0 | 0 | 0 |
| Median (IQR) | 66 (59-72) | 70 (63-76) | 61 (55-67) | 60 (55-69) | 70 (64-76) | 69 (59-73) | 65 (56-72) | 64 (56-66) |
| Gender |  |  |  |  |  |  |  |  |
| Female | 268 | 62 | 57 | 19 | 240 | 20 | 63 | 15 |
| Male | 235 | 65 | 60 | 66 | 202 | 18 | 53 | 51 |
| Smoking history |  |  |  |  |  |  |  |  |
| Yes | 69 | 104 | 61 | / | 335 | 27 | 101 | 39 |
| No | 420 | 23 | 56 | / | 33 | 9 | 12 | 27 |
| NA | 14 | 0 | 0 | / | 74 | 2 | 3 | 0 |
| T stage |  |  |  |  |  |  |  |  |
| T0 and T1 | 167 | 43 | 54 | 71 | / | 10 | / | 16 |
| T2 | 271 | 82 | 50 | 12 | / | 22 | / | 30 |
| T3 | 44 | 2 | 8 | 2 | / | 3 | / | 16 |
| T4 | 18 | 0 | 5 | 0 | / | 2 | / | 4 |
| TX | 3 | 0 | 0 | 0 | / | 1 | / | 0 |
| N stage |  |  |  |  |  |  |  |  |
| N0 | 328 | 94 | 87 | 82 | / | 37 | / | 14 |
| N1 | 92 | 33 | 8 | 3 | / | 0 | / | 32 |
| N2 | 68 | 0 | 22 | 0 | / | 0 | / | 20 |
| N3 | 2 | 0 | 0 | 0 | / | 0 | / | 0 |
| NX | 12 | 0 | 0 | 0 | / | 1 | / | 0 |
| NA | 1 | 0 | 0 | 0 | / | 0 | / | 0 |
| M stage |  |  |  |  |  |  |  |  |
| M0 | 334 | 127 | 117 | 85 | / | 38 | / | 66 |
| M1 | 24 | 0 | 0 | 0 | / | 0 | / | 0 |
| MX | 140 | 0 | 0 | 0 | / | 0 | / | 0 |
| NA | 5 | 0 | 0 | 0 | / | 0 | / | 0 |
| OS |  |  |  |  |  |  |  |  |
| Alive | 322 | 76 | 68 | 40 | 298 | 10 | 49 | / |
| Dead | 181 | 51 | 49 | 45 | 122 | 28 | 66 | / |
| NA | 0 | 0 | 0 | 0 | 22 | 0 | 1 | / |
| Median (IQR) | 22.6 (14.5-38.3) | 52.4 (22.9-68.9) | 68.0 (40.4-84.3) | 68.0 (25.0-113.0) | 27.5 (18.0-33.7) | 70.8 (22.9-132.3) | 33.0 (13.5-59.2) | / |
| RFS |  |  |  |  |  |  |  |  |
| Non-relapse | 208 | 87 | 58 | 58 | / | / | / | 28 |
| Relapse | 90 | 37 | 58 | 27 | / | / | / | 38 |
| NA | 205 | 3 | 1 | 0 | / | / | / | 0 |
| Median (IQR) | 20.9 (14.0-38.2) | 43.3 (16.2-64.2) | / | 62.0 (18.0-107.0) | / | / | / | 23.1 (15.1-38.8) |
