# Supplementary material for: Discovery of a novel ROS-based signature for predicting prognosis and immunosuppressive tumor microenvironment in lung adenocarcinoma
Source: J Cancer. 2024 Mar 17;15(9):2691–711. doi: 10.7150/jca.93975 (PMC10988302; doi:10.7150/jca.93975)
Supplement: Supplementary file 2 — Supplementary tables. [file jcav15p2691s2.zip › final_tables_20240312/Table S7_Multivariate Cox regression for patient outcome in training and validation cohorts.docx]

**Table S7. Multivariate Cox regression for patient outcome in training and validation cohorts.**

| **Characteristics** | **TCGA-LUAD** | | **GSE50081** | | **GSE13213** | | **GSE29016** | | **LUAD cohort^*^** | |
| --- | --- | --- | --- | --- | --- | --- | --- | --- | --- | --- |
|  | **HR (95% CI)** | ***P* value** | **HR (95% CI)** | ***P* value** | **HR (95% CI)** | ***P* value** | **HR (95% CI)** | ***P* value** | **HR (95% CI)** | ***P* value** |
| Age |  | 0.41 |  | 0.45 |  | 0.22 |  | 0.67 |  | 0.16 |
| < 60 | 1 |  | 1 |  | 1 |  | 1 |  | 1 |  |
| ≥ 60 | 1.2 (0.8-1.6) |  | 1.5 (0.5-4.4) |  | 1.5 (0.8-2.8) |  | 1.3 (0.4-4.2) |  | 1.9 (0.8-4.8) |  |
| Gender |  | 0.81 |  | 0.26 |  | 0.13 |  | 0.97 |  | 0.77 |
| Female | 1 |  | 1 |  | 1 |  | 1 |  | 1 |  |
| Male | 1.0 (0.7-1.3) |  | 1.4 (0.8-2.5) |  | 1.9 (0.8-4.1) |  | 1.0 (0.4-2.7) |  | 0.8 (0.2-2.8) |  |
| Smoking history |  | 0.68 |  | 0.64 |  | 0.64 |  | 0.47 |  | 0.96 |
| No | 1 |  | 1 |  | 1 |  | 1 |  | 1 |  |
| Yes | 1.1 (0.7-1.8) |  | 1.2 (0.5-2.8) |  | 0.8 (0.4-1.9) |  | 1.5 (0.5-4.6) |  | 1.0 (0.4-2.9) |  |
| T stage |  |  |  |  |  |  |  |  |  |  |
| T0 and T1 | 1 |  | 1 |  | 1 |  | 1 |  | 1 |  |
| T2 | 1.2 (0.8-1.7) | 0.48 | 1.7 (0.8-3.5) | 0.18 | 1.4 (0.7-2.6) | 0.33 | 0.4 (0.2-1.1) | 0.07 | 1.2 (0.4-3.3) | 0.72 |
| T3 | 2.4 (1.4-4.2) | < 0.01 | 9.3 (1.4-61.0) | 0.02 | 1.3 (0.4-4.1) | 0.62 | 0.9 (0.2-5.0) | 0.90 | 1.3 (0.4-4.2) | 0.67 |
| T4 | 2.0 (0.9-4.6) | 0.09 | / |  | 3.1 (1.0-9.5) | 0.05 | 4.6 (0.7-30.8) | 0.12 | 2.4 (0.5-12.2) | 0.31 |
| TX | 4.2 (0.5-34.2) | 0.18 | / |  | / |  | 1.2 (0.1-12.7) | 0.91 | / |  |
| N stage |  |  |  | 0.03 |  |  |  | 0.07 |  |  |
| N0 | 1 |  | 1 |  | 1 |  | 1 |  | 1 |  |
| N1 | 2.4 (1.6-3.5) | < 0.001 | 2.0 (1.1-3.7) |  | 1.6 (0.5-4.8) | 0.39 | / |  | 1.6 (0.5-5.0) | 0.39 |
| N2 | 2.0 (1.4-3.1) | < 0.001 | / |  | 3.7 (1.9-7.3) | < 0.01 | / |  | 4.3 (1.3-13.8) | 0.01 |
| N3 | Inf | 0.99 | / |  | / |  | / |  | / |  |
| NX | 1.0 (0.2-4.1) | 0.98 | / |  | / |  | 8.3 (0.8-83.9) |  | / |  |
| M stage |  | 0.020 |  | / |  | / |  | / |  | / |
| M0 | 1 |  |  |  |  |  |  |  |  |  |
| M1 | 1.8 (1.0-3.4) | 0.07 |  |  |  |  |  |  |  |  |
| MX | 1.2 (1.0-1.7) | 0.48 |  |  |  |  |  |  |  |  |
| Risk group |  | < 0.01 |  | 0.03 |  | < 0.01 |  | < 0.01 |  | < 0.01 |
| Low-risk | 1 |  | 1 |  | 1 |  | 1 |  | 1 |  |
| High-risk | 2.9 (2.0-4.1) |  | 2.0 (1.1-3.6) |  | 4.4 (1.9-10.0) |  | 3.8 (1.4-10.2) |  | 2.7 (1.3-5.6) |  |

*: Cox regression for PFS in the Chinese LUAD cohort
